# HELLS controls mitochondrial dynamics and genome stability in liver cancer by collusion with MIEF1

**DOI:** 10.1038/s41419-025-07589-x

**Published:** 2025-04-02

**Authors:** Sung Kyung Choi, Jihye Park, Sang Yun Ha, Myoung Jun Kim, Seor I Ahn, Jeongah Kim, Woong Sun, Yeong Min Park, Suk Woo Nam, Jeung-Whan Han, Keunsoo Kang, Jueng Soo You

**Affiliations:** 1 School of Medicine, Konkuk University, Chungju, 27478 Korea; 2 College of Natural Sciences, Dankook University, Cheonan, 31116 Korea; 3 Department of Pathology and Translational Genomics, Samsung Medical Center, Sungkyunkwan University School of Medicine, Seoul, 06351 Korea; 4 Department of Anatomy, College of Medicine, Korea University, Seoul, 02841 Korea; 5 Department of Integrative Biological Sciences and Industry, Sejong University, Seoul, 05006 Korea; 6 Department of Pathology, College of Medicine, Catholic University, Seoul, 06649 Korea; 7 Research Center for Epigenome Regulation, School of Pharmacy, Sungkyunkwan University, Suwon, 16419 Korea; 8 Research Institute of Medical Science, KU Open Innovation Center, Chungju, 27478 Korea

**Keywords:** Cancer epigenetics, Chromatin remodelling, Mechanisms of disease

## Abstract

Dysregulated chromatin remodelers have emerged as critical disease targets. However, owing to the pleiotropic functions of chromatin remodelers, the underlying mechanisms of their effects on cancer have been difficult to elucidate. Here, we investigated the helicase lymphoid-specific (HELLS) oncogenic mechanism by identifying a new direct transcriptional target. Using loss or gain experiments, we identified Mitochondrial elongation factor 1 (MIEF1) as a critical target of the HELLS molecular network in liver cancer. Liver cancer patients with a poor prognosis exhibited upregulated expression of MIEF1, and MIEF1 knockdown led to the loss of tumor capabilities, indicating MIEF1 as an oncogene in liver cancer. Suppressing the HELLS-MIEF1 axis caused mitochondrial hyperfusion, energy deprivation, and further resulting senescence. HELLS knockdown globally increased histone 3 lysine 9 trimethylation (H3K9me3), especially in genomic hotspots with upregulation of SUV39H1 and further augmented DNA methylation. This stabilized genome and hyperfused mitochondria led to reduced levels of reactive oxygen species (ROS) and DNA damage. Finally, tumor cells became famished and calm. We further validated the functions of the HELLS-MIEF1 axis by MIEF1 overexpression and mitochondrial fusion drug. Our study has important implications for medical science by highlighting the crosstalk between epigenetics and metabolism through nuclear chromatin remodeler HELLS and mitochondrial protein MIEF1.

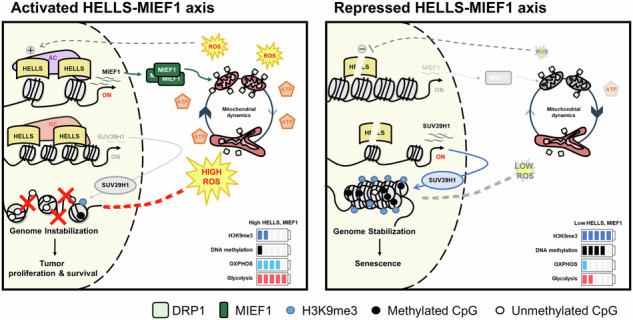

## Introduction

Chromatin is a highly dynamic structure influenced by DNA methylation, histone modifications, nucleosome occupancy, and noncoding RNAs [1, 2]. Chromatin accessibility directly affects transcriptional potential and various critical processes determining cell fate [3]. Chromatin remodelers are crucial for dynamic accessibility change using ATP and other epigenetic modulators [4]. Interestingly, these ATP-dependent chromatin remodelers have exhibited high mutation rates and abnormal expression in malignant cells [5].

Lymphoid-specific helicase (HELLS), a member of the SNF2-like chromatin remodeler family, is highly conserved and distinctively presents as a monomeric protein, unlike other chromatin remodeling ATPases [6]. HELLS knockout mice exhibit reduced birth weight and multiple organ defects and die shortly after birth, underscoring its essential role in normal development [7, 8]. Mutations in HELLS are linked to immunodeficiency, centromeric region instability, and facial anomalies (ICF) syndrome [9–11]. HELLS interacts with various epigenetic modulators, such as DNA methyltransferases (DNMTs), ten-eleven-translocation (TET) proteins, histone methyltransferase G9a, and histone deacetylase (HDAC), to regulate the comprehensive epigenetic landscape [6, 12–16]. Therefore, exploring how HELLS modulates its targets and contributes to the epigenetic landscape in specific contexts is of great importance and priority.

Mitochondria are pivotal for cellular energy production and play essential roles in cell survival and death [17]. Their morphology, which often determines their function, is dynamically regulated through cycles of fusion and fission [18]. Fusion, mediated by proteins such as optic atrophy 1 (OPA1) and mitofusin 1 and 2, results in elongated tubular mitochondria [19]. Conversely, fission, driven by dynamin-related protein 1 (DRP1) and its receptors—fission protein 1, mitochondrial fission factor, and mitochondrial dynamics proteins 49 kDa and 51 kDa—leads to fragmented mitochondria, which are crucial for mitochondrial biogenesis and clearance [20]. Mitochondrial fusion and fission imbalances have been known to induce pathological states by impacting cellular metabolism, proliferation, and differentiation [21].

In this study, we identified mitochondrial elongation factor 1 (MIEF1), one of the DRP1 receptors, as a novel oncogene and a direct transcriptional target and critical executor downstream of HELLS in liver cancer. HELLS knockdown induced mitochondrial hyperfusion and depleted cellular energy by downregulating MIEF1, leading to cellular senescence. Suppression of the HELLS-MIEF1 axis increased the global level of H3K9me3 and DNA methylation, especially in hot spots, accompanied by reduced reactive oxygen species (ROS) and DNA damage, ultimately stabilizing the genome. Therefore, we strongly propose that the HELLS–MIEF1 axis regulates the oncogenic signature in liver cancer by modulating cellular energy and genomic stability. These findings further offer new insights into the active crosstalk between the nucleus and mitochondria and could advance the development of novel cancer therapeutics.

## Materials and methods

### Cell culture

The human cell lines were obtained from KCLB (Korean Cell Line Bank) and maintained in our laboratory. All cell lines were periodically tested for mycoplasma contamination. Each cell line was maintained in DMEM and RPMI1640 (Welgene) supplemented with 10% FBS (Welgene) and 100 units/mL of penicillin-streptomycin (Invitrogen). All cells were cultured at 37 °C in a humidified incubator with 5% CO_2_. Cells were treated with Chaetocin (Abcam, Cat# ab144534) at a final concentration of 50 µM for 24 h and with BGP-15 (Cayman Chemical, Cat# 17503) at a final concentration of 50 µM for 4 h.

### shRNA infection

shHELLS and shMIEF1 constructs were purchased from Sigma-Aldrich. For lentivirus production, the MISSION lentiviral packaging mix was used according to the manufacturer’s protocol. Infected derivative cells stably expressing shRNA were selected in the presence of 1.25 mg/mL puromycin.

Additional materials and methods are described in the Supporting Materials and Methods.

## Results

### HELLS, a nuclear chromatin remodeler, directly targets mitochondrial protein MIEF1 in liver cancer

HELLS expression is elevated in liver tumor tissues compared to normal samples in the ICGC (International Cancer Genome Consortium), and high levels were consistently associated with poor prognosis in both ICGC data and patient samples, consistent with a recent report [22] (Supplementary Fig. S1A–C). The non-tumorigenic liver cell line MIHA showed relatively low levels of HELLS expression, whereas HCC cell lines, including HepG2, SNU398, Huh7, and Hep3B, exhibited increased HELLS expression (Supplementary Fig. S1D). Knockdown of HELLS using two shRNAs significantly diminished cell proliferation, wound healing capacity, and colony formation (Supplementary Fig. S1E–H), underscoring the oncogenic role of HELLS in HCC.

**Fig. 1:**
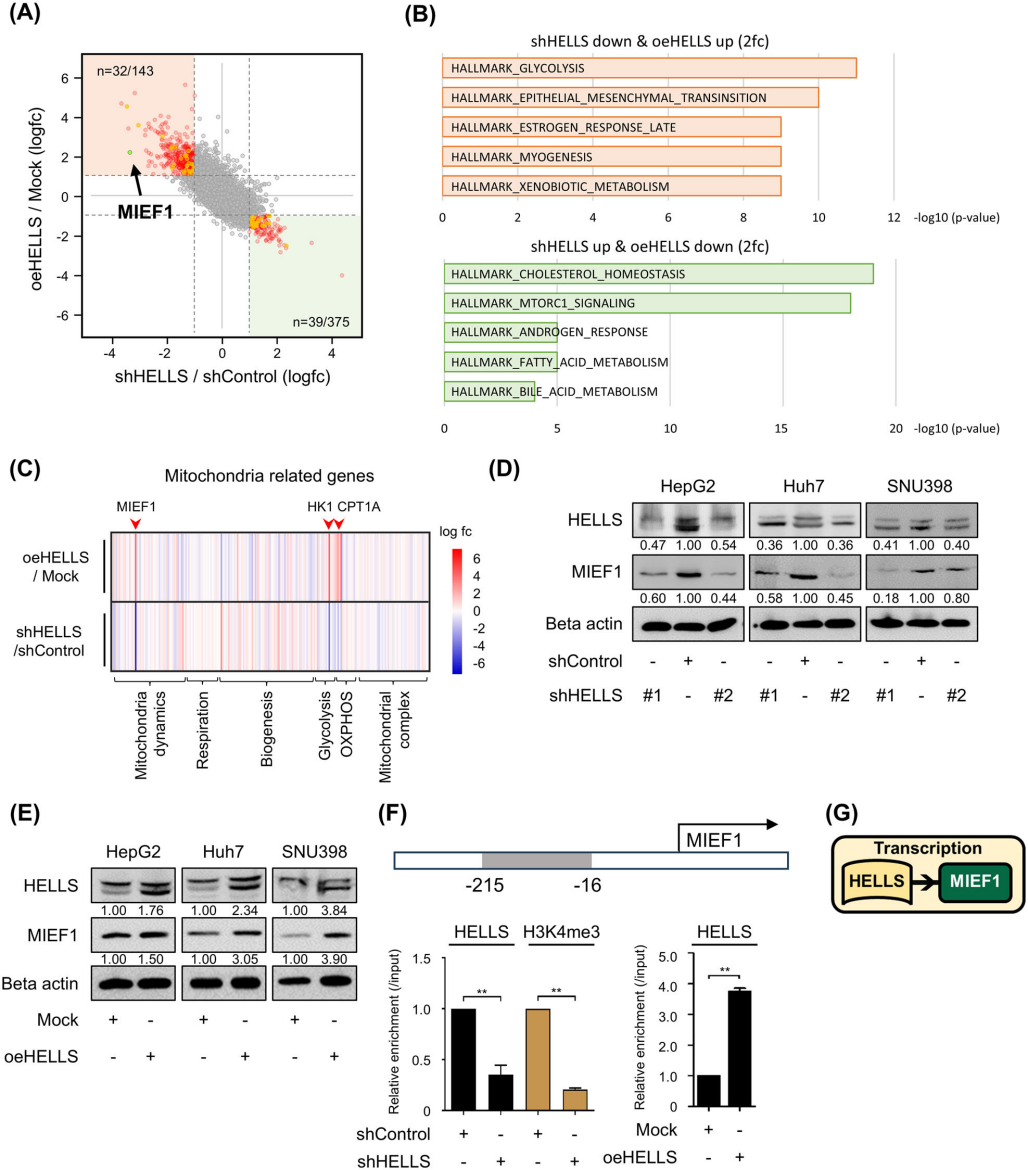
HELLS targets the mitochondrial protein MIEF1. **A** HELLS-dependent transcriptome analysis. Red, yellow, and green dots indicate significant changes in differentially expressed genes (DEGs) with a 2-fold change and 0.05 *p*-value cutoffs. Red dots represent DEGs (*n* = 143 and 375). Yellow dots represent genes positively correlated with HELLS expression in UALCAN (*n* = 32, 39). The green dot highlights MIEF1 (included in both red and yellow categories). **B** HELLS Hallmark analysis of HELLS positive genes (downregulated by HELLS knockdown and upregulated by HELLS overexpression, #143) and HELLS negative genes (upregulated by HELLS knockdown and downregulated by HELLS overexpression, # 375). **C** The expression changes of mitochondria-related gene sets upon the loss and gain of HELLS. **D**, **E** Protein levels of HELLS, MIEF1, and Beta actin upon HELLS depletion/overexpression in HepG2, Huh7, and SNU398 cells were measured using western blots. **F** Schematic of the MIEF1 promoter PCR region (Top), the enrichments of HELLS and H3K4me3 at the MIEF1 promoter in Huh7 cells were measured using ChIP-qPCR (mean ± S.E.M., **p < 0.01, *n* = 3). **G** The graphic model summarizes the key findings illustrated in Fig. 1.

To elucidate HELLS’ downstream targets, we conducted transcriptome analyses under both loss- and gain-of-function conditions. We identified 143 genes as positive targets (downregulated upon HELLS knockdown and upregulated upon HELLS overexpression) and 375 genes as negative targets (upregulated upon HELLS knockdown and downregulated upon HELLS overexpression, Supplementary Fig. S2A). HELLS-positive target genes were enriched in pathways related to glycolysis, oxidative phosphorylation, and the reactive oxygen species, indicating a profound impact on cellular metabolic processes and oxidative stress regulation. Conversely, HELLS-negative genes showed significant enrichment in cholesterol homeostasis, G2M checkpoint, and E2F targets (Fig. 1A, B, Supplementary Fig. S2B). KEGG analysis and Gene set enrichment analysis (GSEA) further revealed a significant enrichment of metabolic pathways upon loss and gain of HELLS, indicating a potential regulatory role of HELLS in cellular metabolism and cancer progression (Supplementary Fig. S2C and D).

Based on the transcriptome analyses, we hypothesize that HELLS plays a critical regulatory role in cellular metabolism and mitochondrial function. We then analyzed the expression changes in mitochondrial-related genes upon HELLS modulation. Among the genes categorized into six subgroups, MIEF1, HK1, and CPT1A emerged as particularly prominent (Fig. 1C). Further analysis using the UALCAN database suggested that MIEF1, identified as a mitochondrial outer membrane protein and DRP1 adapter known for inducing fission, emerged as a putative direct target of HELLS, suggesting its potential role in mitochondrial dynamics and cellular metabolism (Fig. 1A). MIEF1 levels were downregulated upon HELLS loss and, conversely, upregulated upon HELLS gain (Fig. 1D, E, and Supplementary Fig. S3A). Furthermore, we showed that HELLS binds to the MIEF1 promoter by chromatin immunoprecipitation (ChIP) qPCR. This binding is dependent on HELLS expression and accompanied by changing histone marks (Fig. 1F and Supplementary Fig. S3B), signifying that MIEF1 is a direct transcriptional target of HELLS in HCC (Fig. 1G).

### MIEF1 is a putative oncogene in liver cancer

**Fig. 2:**
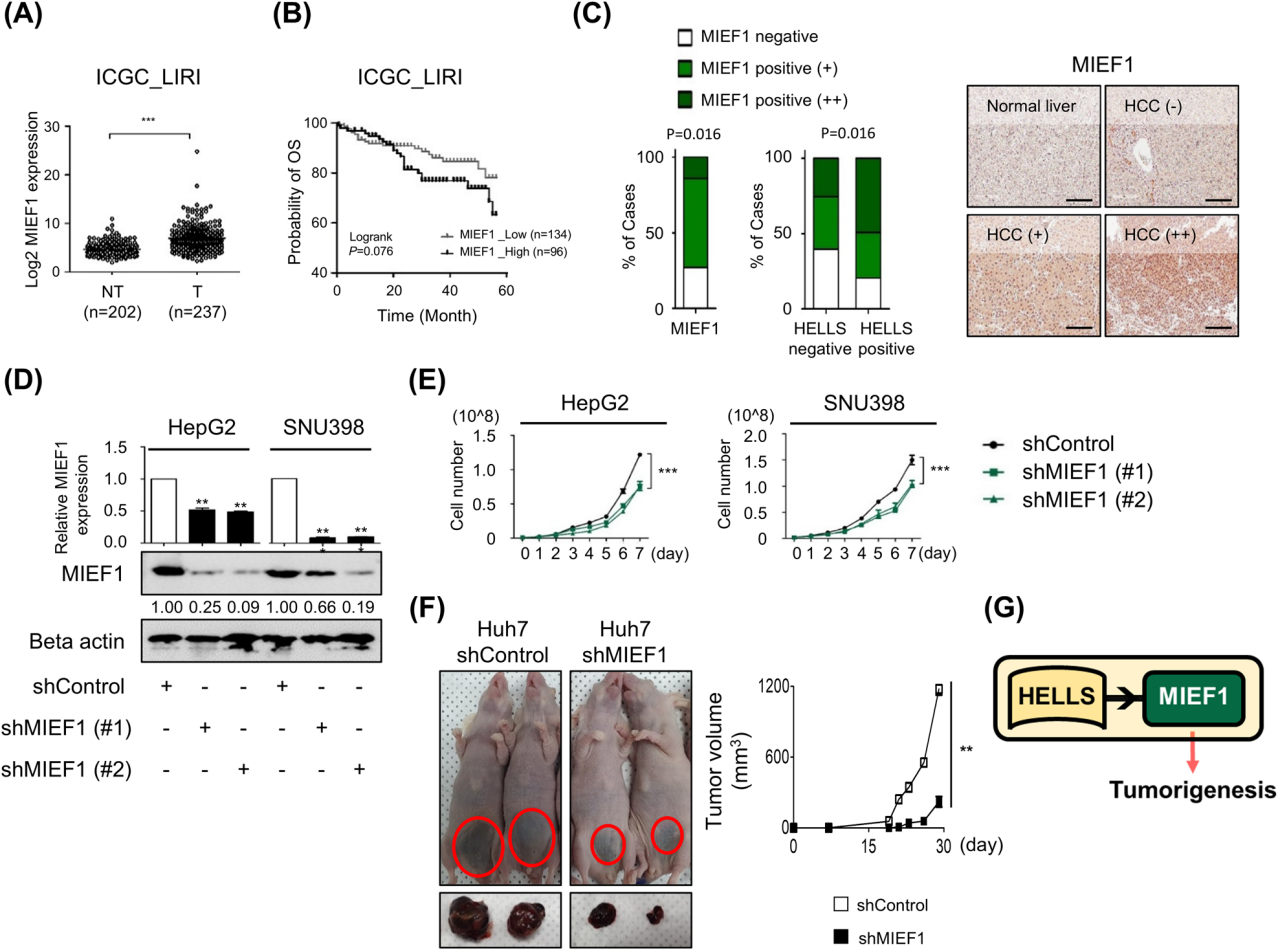
MIEF1 has an oncogenic function in liver cancer. **A** MIEF1 expression in non-tumor (NT) and tumor (T) tissues presented as mean ± S.D. (***p < 0.001). Horizontal lines are the median. **B** Kaplan–Meier survival analysis on MIEF1 levels. The *p*-value was calculated using the log-rank test. **C** MIEF1 expression in liver cancer tissues. MIEF1 protein levels were analyzed in total liver cancer tissue (left panel) and further stratified based on HELLS expression levels (right panel) using bar charts. Representative Immunohistochemistry (IHC) staining images from each HELLS expression intensity level were included: negative (−), positive (+), and strong (++). The scale bar represents 100 μm. **D** MIEF1 expression levels upon MIEF1 depletion. The relative mRNA levels of MIEF1 in HepG2 and SNU398 cells were presented as mean ± S.E.M. (**p < 0.01, *n* = 3). The protein levels of MIEF1 and Beta actin in HepG2 and SNU398 cells were measured upon MIEF1 depletion using western blots. **E** Growth rates of upon MIEF1 loss. Cell numbers were presented as mean ± S.E.M. in HepG2 and SNU398 cells (***p < 0.001, *n* = 3). **F** Tumor growth in xenograft models. Tumor volumes were measured using nude mice injected with Huh7 shControl and shMIEF1 cells (*n* = 4 per group) and presented as mean ± S.E.M. (**p < 0.01). **G** The schematic model summarizes the key findings illustrated in Fig. 2.

To explore the role of MIEF1 in liver cancer, we analyzed its expression using ICGC data. MIEF1 expression was significantly higher in liver tumors compared to normal tissues, and higher MIEF1 levels were associated with poorer prognosis in patients. The statistical insignificance of patient survival analyses between MIEF1_High and MIEF1_Low may be due to the antibody’s efficacy in our experience (Fig. 2A, B). Immunohistochemical analysis further confirmed that MIEF1 expression was markedly elevated in hepatocellular carcinoma (HCC) tissues compared to normal liver tissues—elevated MIEF1 levels correlated with reduced survival rates in HCC patients. Additionally, the proportion of MIEF1-positive cells was substantially higher in HELLS-positive tissues than in HELLS-negative tissues, indicating a positive correlation between HELLS and MIEF1 expression (Fig. 2C and Supplementary Fig. S4A). To determine the specific role of MIEF1, we conducted a loss of function study by generating stable MIEF1 knockdown HCC cell lines using shRNAs (Fig. 2D). MIEF1 knockdown mimicked the phenotypic effects of HELLS depletion, resulting in reduced cell proliferation, impaired wound healing, and decreased colony forming ability (Fig. 2E and Supplementary Fig. S4B and C). In vivo, MIEF1 knockdown significantly reduced tumor size and weight, while the overall body weight of the mouse models remained unchanged (Fig. 2F and Supplementary Fig. S4D–F). Furthermore, overexpression of HELLS increased MIEF1 levels and enhanced colony-forming ability in non-tumorigenic liver cancer (Supplementary Fig. S4G and H). These findings suggest that MIEF1 acts as a novel oncogene in liver cancer, contributing to tumor growth and poor prognosis (Fig. 2G).

### HELLS controls mitochondrial dynamics and cellular energy levels through MIEF1

**Fig. 3:**
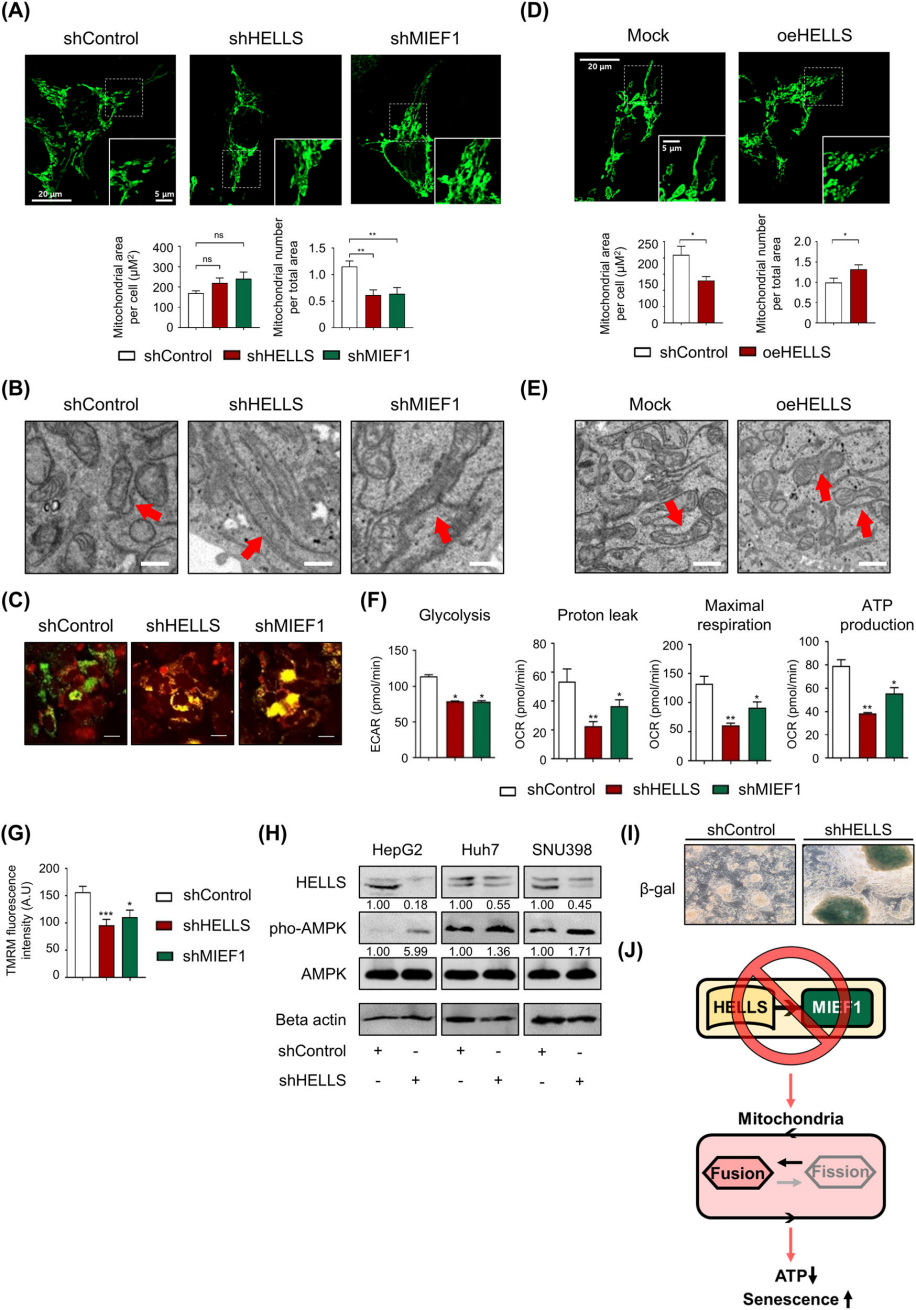
HELLS regulates mitochondrial dynamics and cellular energy homeostasis via MIEF1. **A** Representative confocal images of mitochondria upon HELLS and MIEF1 depletion. HepG2 cells were transfected with mt-ro2GFP. The scale bar represents 20 μm. Quantification of mitochondrial area per cell (μM²) and mitochondrial number per total area were measured upon HELLS and MIEF1 depletion. (mean ± S.E.M., ns non-significant, **P < 0.01, *n* = 3). **B** Representative Transmission electron microscopy (TEM) images of mitochondria upon HELLS and MIEF1 depletion in HepG2. Arrows indicate mitochondria. Scale bars = 10 μm. **C** Representative confocal images of mitochondrial fusion upon HELLS and MIEF1 depletion. HepG2 cells were transfected with mtGreen and mtRed and fused by polyethylene glycol (PEG). The scale bar represents 10 μm. **D** Representative confocal images of mitochondria upon HELLS overexpression. HepG2 cells were transfected with mt-ro2GFP. The scale bar represents 20 μm. Quantification of mitochondrial area per cell (μM²) and mitochondrial number per total area were measured upon HELLS overexpression. (mean ± S.E.M., *P < 0.05, *n* = 3). **E** Representative TEM images of mitochondria upon HELLS overexpression in HepG2. Mitochondria are indicated by arrows. Scale bars = 10 μm. **F** Seahorse experiment data upon HELLS and MIEF1 depletion. Glycolysis, proton leak, maximal respiration, and ATP production were measured in Huh7 cells (mean ± S.E.M., *P < 0.05, **P < 0.01, *n* = 3). **G** Quantification of TMRM fluorescence intensity upon HELLS and MIEF1 depletion in HepG2 (mean ± S.E.M., *P < 0.05, ***P < 0.001, *n* = 3). **H** Protein levels of HELLS, pho-AMPK, AMPK, and Beta actin upon HELLS depletion in HepG2, Huh7, and SNU398 cells were measured using western blots. **I** Representative images of senescence-associated β-galactosidase (SA-β-gal) staining upon HELLS depletion in HepG2. **J** The schematic model summarizes the key findings illustrated in Fig. 3.

Given that MIEF1, a mitochondrial dynamics protein, was identified as a novel downstream target of HELLS, we investigated the effects of HELLS on mitochondrial morphology and metabolism. Reduction of HELLS expression led to the formation of a tubular mitochondrial network, indicating increased mitochondrial fusion. Similarly, MIEF1 knockdown resulted in elongated mitochondria (Fig. 3A). Although the reduction of HELLS and MIEF1 did not significantly increase the total mitochondrial area, the number of mitochondria decreased, suggesting that the mitochondria became more interconnected. (Fig. 3A and Supplementary Fig. S5A). Transmission electron microscopy (TEM) confirmed that both HELLS and MIEF1 knockdowns induced an elongated, tubular mitochondrial morphology characteristic of the fusion state (Fig. 3B). These findings indicate that both HELLS and MIEF1 are critical regulators of mitochondrial dynamics. GSEA revealed that loss of HELLS resulted in decreased expression of mitochondrial fission genes and increased expression of mitochondrial fusion genes (Supplementary Fig. S5B). To validate these findings, cells with differentially labeled mitochondria were fused and analyzed. Fluorescence imaging showed that the fusion area, represented by yellow mitochondria, was significantly larger in HELLS and MIEF1 knockdown cells compared to shControl cells (Fig. 3C). In contrast, cells overexpressing HELLS displayed increased mitochondrial fragmentation. The decrease in total mitochondrial area and an increase in mitochondrial number support the conclusion of enhanced fragmentation. (Fig. 3D and Supplementary Fig. S5C). TEM analysis further demonstrated that overexpression of HELLS resulted in mitochondria adopting a peanut-shaped morphology, indicative of enhanced mitochondrial fission (Fig. 3E). We found that the depletion of HELLS and MIEF1 significantly impacted mitochondrial function, as evidenced by reduced glycolysis rate, proton leak, maximal respiration, and ATP production (Fig. 3F). Further investigation revealed that the knockdown of HELLS and MIEF1 caused a significant decrease in membrane potential, as indicated by reduced TMRM fluorescence (Fig. 3G). This reduction in membrane potential underscores the critical role of the HELLS-MIEF1 axis in maintaining mitochondrial integrity and function. Transcriptome analysis confirmed that gene sets involved in glycolysis, oxidative phosphorylation (OXPHOS), and mitochondrial complex III, all essential for ATP production, were downregulated following HELLS knockdown and upregulated with HELLS overexpression (Supplementary Fig. S5D and E). Moreover, HELLS knockdown resulted in elevated levels of phosphorylated AMP-activated protein kinase (AMPK) (Fig. 3H). Suppression of the HELLS-MIEF1 axis led to mitochondrial hyperfusion, energy depletion, and subsequent cellular senescence (Fig. 3I), indicating that the HELLS-MIEF1 axis is essential for cancer cell survival by modulating mitochondrial dynamics (Fig. 3J).

### HELLS depletion leads to global heterochromatin formation via increased H3K9me3

**Fig. 4:**
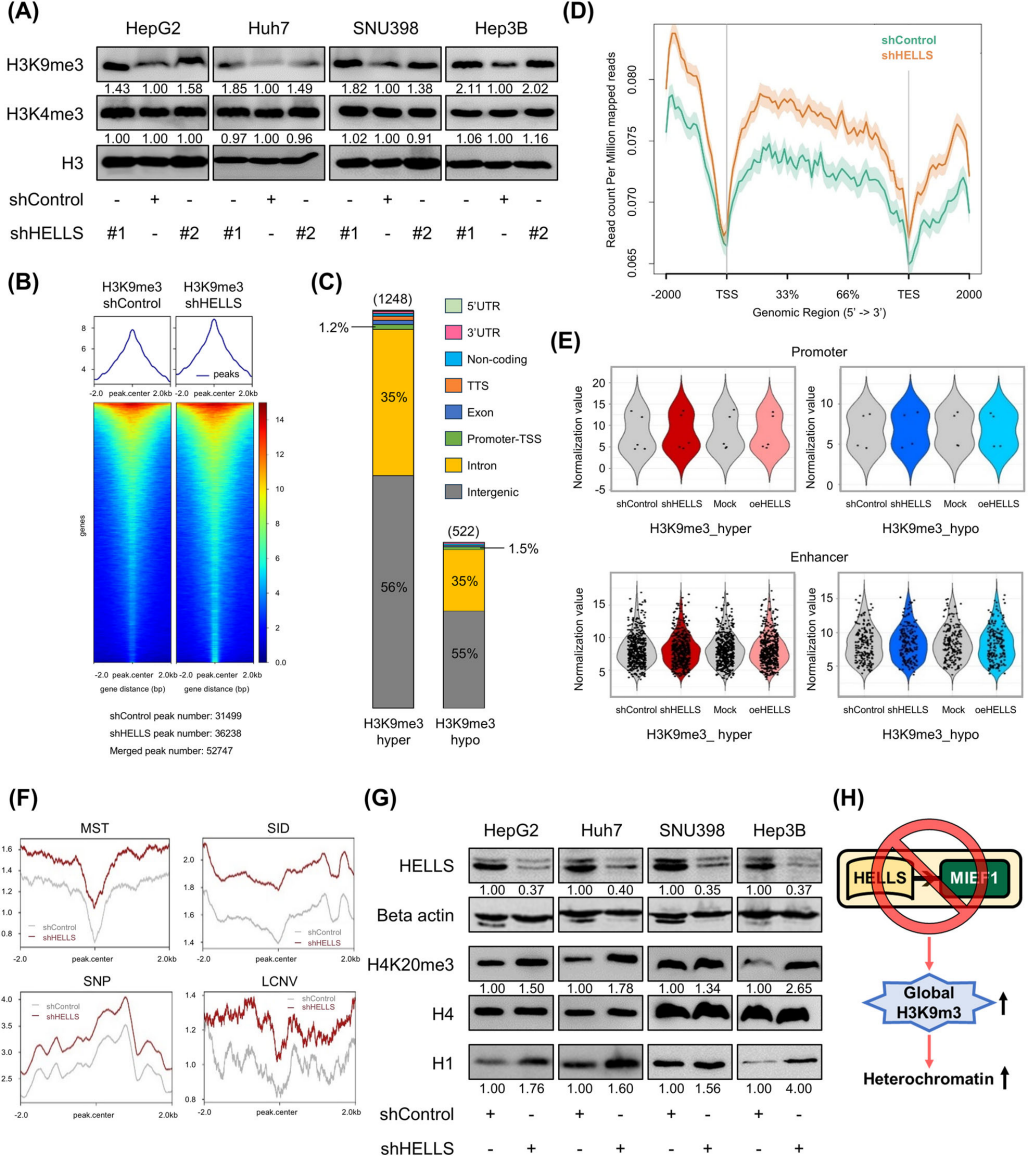
Decreased HELLS induces global heterochromatin with the increased global level of H3K9me3. **A** Protein levels of H3K9me3, H3K4me3, and Histone H3 upon HELLS depletion in HepG2, Huh7, SNU398, and Hep3B cells were measured using western blots. **B** ChIP-seq profiles showed the alterations in distribution and intensity of H3K9me3 peaks upon HELLS depletion in HepG2 cells. **C** Genomic distribution of differential H3K9me3 peaks upon HELLS depletion with log2 fold change (log2FC) ≥ 1 in HepG2 cells. **D** H3K9me3 enrichment signal across genomic regions upon HELLS depletion in HepG2 cells. **E** Gene expression levels from microarray analysis of genes associated with hypermethylated and hypomethylated H3K9me3 peaks at promoters and enhancers upon HELLS depletion in HepG2 cells. **F** Distribution of H3K9me3 peaks across various genomic hotspots upon HELLS depletion in HepG2 cells. The hotspots include microsatellites (MST), small indels (SID), single nucleotide polymorphisms (SNP), and long copy number variations (LCNV). **G** Protein levels of HELLS, Beta actin, H4K20me3, Histone H4, and Histone H1 upon HELLS depletion in HepG2, Huh7, SNU398, and Hep3B cells were measured using western blots. **H** The schematic model summarizes the key findings illustrated in Fig. 4.

Next, we investigated the changes in chromatin structure mediated by HELLS, accompanied by alterations in histone modifications. Of note, we found that loss of HELLS expression significantly increased H3K9me3 levels among various histone modifications (Supplementary Fig. S6A and B). This increase in H3K9me3 levels was consistently observed across four HELLS knockdown cell lines (Fig. 4A). Similarly, MIEF1 knockdown also resulted in increased H3K9me3 levels, while overexpression of HELLS led to a decrease in H3K9me3 levels (Supplementary Fig. S6C and D), indicating that the HELLS-MIEF1 axis regulates the global levels of H3K9me3. Additionally, we confirmed that the HELLS-MIEF1 axis modulates H3K9me3 levels in non-tumorigenic MIHA cells (Supplementary Fig. S6E and F). ChIP sequencing analysis revealed that HELLS knockdown globally increased both the number and intensity of H3K9me3 enrichment peaks (Fig. 4B). Using a > 2-fold change criterion, we identified 1,248 hyper-enriched peaks and 522 hypo-enriched peaks. Most of the differential peaks were located in intergenic regions rather than promoter regions (−1000 to +100 bp) (Fig. 4C). The accumulation of H3K9me3 was prominent except at transcription start sites (TSSs) (Fig. 4D). The number of peaks at promoters and enhancers was small and no significant expression changes in associated genes were observed (Fig. 4E and Supplementary Fig. S6G). Notably, enrichment of H3K9me3 at several genomic hotspots was observed in HELLS knockdown cells (Fig. 4F), indicating that loss of HELLS expression leads to more stable chromatin states. These data suggest that accumulation of H3K9me3 due to HELLS depletion likely confers a global heterochromatin state, supported by increases in other heterochromatin markers such as H4K20me3 and linker histone H1 (Fig. 4G, H).

### HELLS knockdown upregulates SUV39H1

**Fig. 5:**
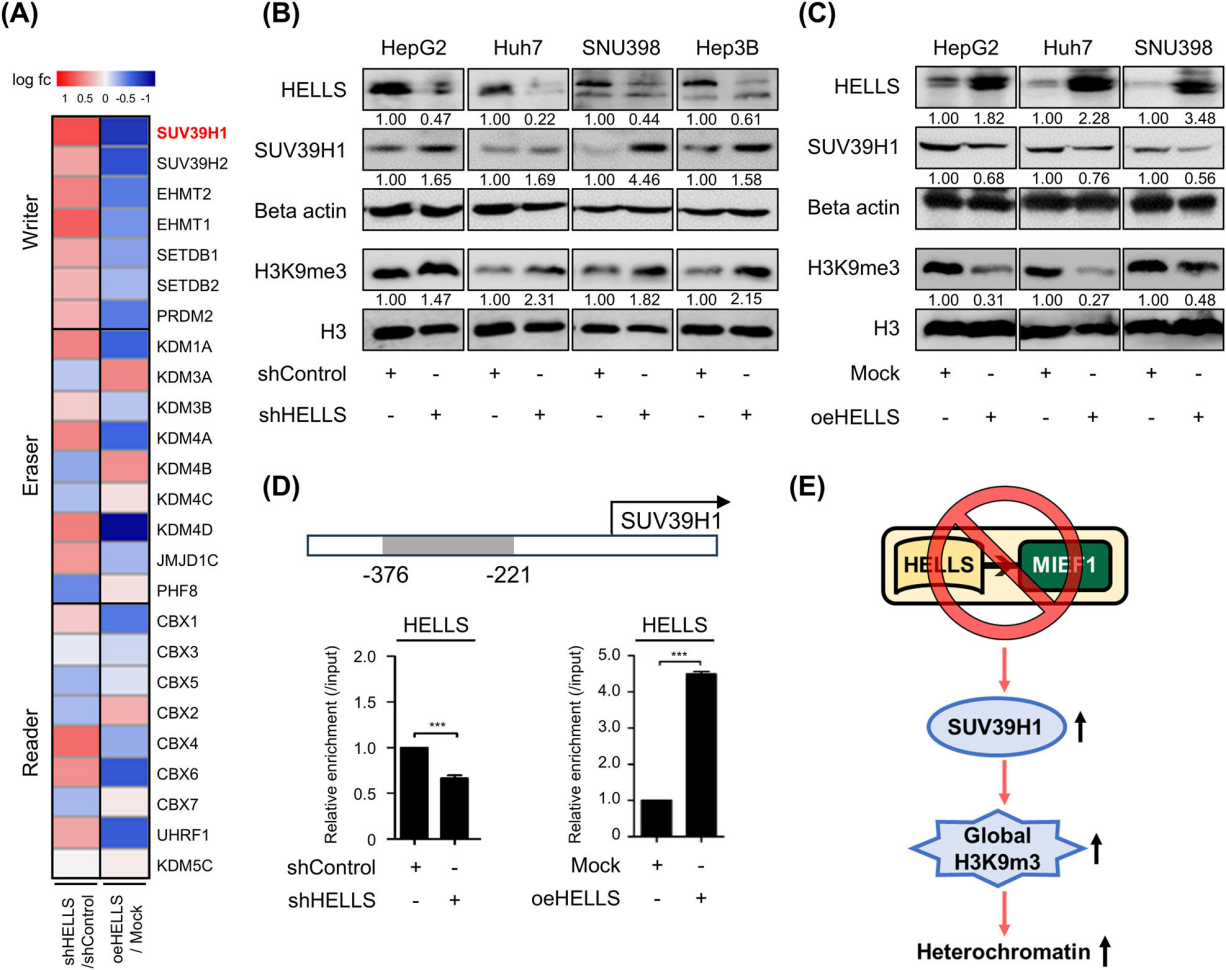
Loss of HELLS induces an increase in SUV39H1 expression. **A** The relative expression levels of the H3K9me3-related gene set upon HELLS depletion and overexpression in HepG2. **B** Protein levels of HELLS, SUV39H1, Beta actin, H3K9me3, and Histone H3 upon HELLS depletion in HepG2, Huh7, SNU398, and Hep3B cells were measured using western blots. **C** Protein levels of HELLS, SUV39H1, Beta actin, H3K9me3, and Histone H3 upon HELLS overexpression in HepG2, Huh7, and SNU398 cells were measured using western blots. **D** HELLS binding at the SUV39H1 promoter. Schematic diagram of the SUV39H1 proximal promoter region (top). The enrichment of HELLS at the SUV39H1 promoter in Huh7 cells was presented using ChIP-qPCR (mean ± S.E.M., ***P < 0.001, *n* = 3). **E** The schematic model summarizes the key findings illustrated in Fig. 5.

To investigate how HELLS knockdown increases H3K9me3, we analyzed the expression of the H3K9me3-related gene set, including writers, erasers, and readers following HELLS knockdown. Among these, SUV39H1, a H3K9 methyltransferase, was notably upregulated in response to HELLS knockdown and downregulated upon HELLS overexpression (Fig. 5A). SUV39H1 expression and H3K9me3 levels were increased upon HELLS loss, and decreased upon HELLS overexpression (Fig. 5B, C and Supplementary Fig. S7A, B). ChIP qPCR results validated SUV39H1 as a transcriptional target of HELLS (Fig. 5D), demonstrating that the upregulation of SUV39H1 is responsible for the increased H3K9me3 levels observed upon HELLS loss. Further supporting this observation, treatment with chaetocin, a specific inhibitor of SUV39H1 [23], effectively reversed the elevated levels of H3K9me3 and other heterochromatin markers, including H4K20me3 and H1, observed in HELLS-depleted cells (Supplementary Data S7C). These findings provide compelling evidence that SUV39H1 plays a crucial role in mediating the establishment of heterochromatin induced by HELLS depletion (Fig. 5E).

### HELLS loss accumulates DNA methylation and promotes stable chromatin states

**Fig. 6:**
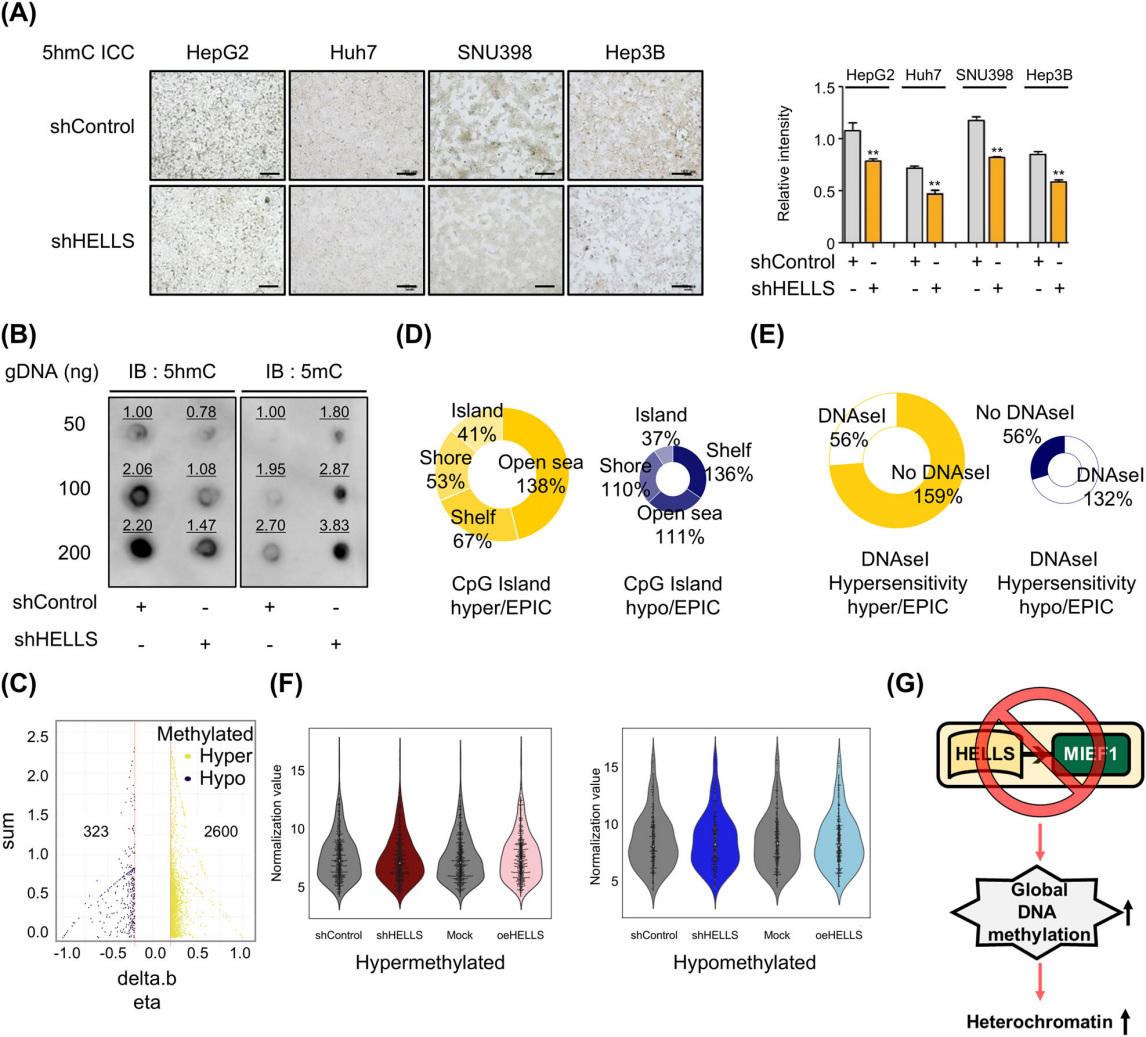
The absence of HELLS promotes DNA methylation accumulation and stabilizes chromatin structures. **A** Representative ICC staining images of 5hmC and quantification of 5hmC levels upon HELLS depletion in HepG2, Huh7, SNU398, and Hep3B cells. The scale bar represents 100 μm. (mean ± S.E.M., **P < 0.01, *n* = 3). **B** Dot blot images of 5hmC and 5mC upon HELLS depletion in HepG2 cells. Quantification values are indicated above each spot, representing relative intensity. **C** HELLS-dependent hypermethylated and hypomethylated DNA sites with beta difference (β diff) ≥│0.2│ in HepG2 cells, measured by Illumina Infinium Methylation EPIC Analysis. **D**, **E** Distribution of hypermethylated and hypomethylated DNA sites within CpG islands/ DNase I hypersensitivity regions upon HELLS depletion in HepG2 cells. **F** Gene expression levels from microarray analysis of genes associated with hypermethylated and hypomethylated DNA methylation sites upon HELLS depletion in HepG2 cells. **G** The schematic model summarizes the key findings illustrated in Fig. 6.

Given the relationship between DNA methylation and HELLS [6, 12, 14, 24–27] and considering that DNA methylation is a well-known molecular feature of heterochromatin with H3K9me3 [28], we investigated global DNA methylation changes. ICC (Immunocytochemistry) and ELISA (Enzyme-Linked Immunosorbent Assay) revealed that HELLS knockdown decreased 5-hydroxymethylcytosine (5hmC) and increased 5-methylcytosine (5mC) levels in four cancer cell lines (Fig. 6A and Supplementary Fig. S8A). These results were validated by dot blot analysis (Fig. 6B). Conversely, HELLS overexpression increased the global level of 5hmC (Supplementary Fig. S8B). To delineate the genomic distribution of the DNA methylation changes, we performed Illumina Infinium Methylation EPIC analysis. We identified 2600 hypermethylated and 323 hypomethylated probes under HELLS knockdown conditions compared to control (delta beta value > 0.2; Fig. 6C). The DNA hypermethylated regions were predominantly located in the open sea (~138%), while the DNA hypomethylated regions were primarily found in the shelf (~136%) (Fig. 6D). Following the loss of HELLS expression, most hypermethylated regions corresponded to “no DNAseI hypersensitivity regions,” indicating already closed chromatin states (Fig. 6E). Similar to H3K9me3 peaks, differential DNA methylation probes did not correlate with local gene expression changes (Fig. 6F), suggesting that HELLS deprivation leads to more stable heterochromatin characterized by increased DNA methylation (Fig. 6G).

### HELLS-MIEF1 axis induces the ROS and unstabilizes the genome

**Fig. 7:**
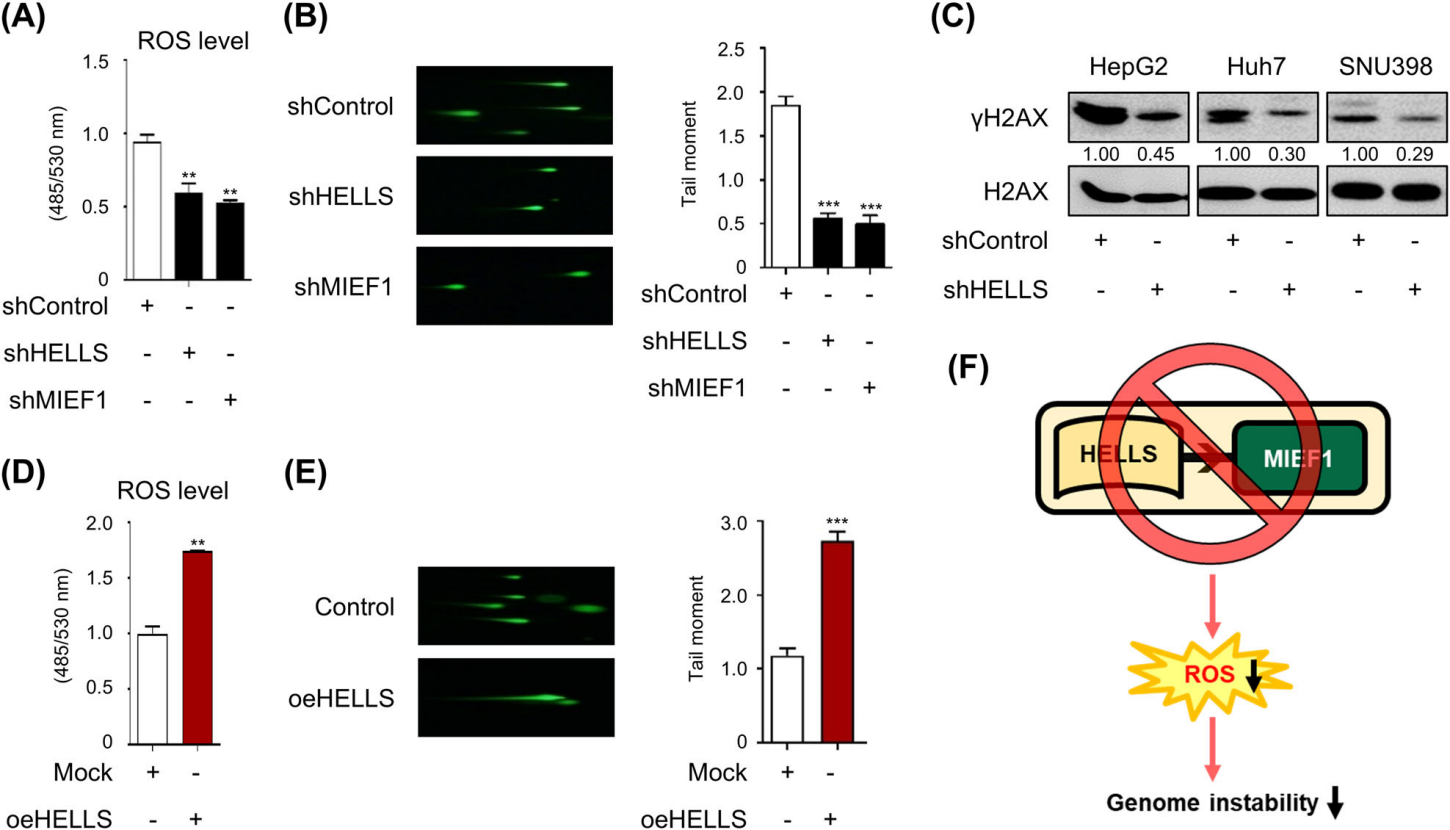
The HELLS-MIEF1 axis promotes ROS production. **A** ROS levels upon HELLS and MIEF1 depletion in HepG2 (mean ± S.E.M., **p < 0.01, *n* = 3). **B** Representative comet images stained with a green DNA staining solution and a bar graph showing the quantification of tail moment upon HELLS and MIEF1 depletion in HepG2 (mean ± S.E.M., ***p < 0.001, *n* = 3). **C** Protein levels of γH2AX and H2AX upon HELLS in HepG2, Huh7, and SNU398 upon HELLS deletion were measured using western blots. **D** ROS levels upon HELLS overexpression in HepG2 (mean ± S.E.M., **p < 0.01, *n* = 3). **E** Representative comet images stained with a green DNA staining solution and a bar graph showing the quantification of tail moment upon HELLS overexpression in HepG2 cells (mean ± S.E.M., ***p < 0.001, *n* = 3). **F** The schematic model summarizes the key findings illustrated in Fig. 7.

Given HELLS’ role as a chromatin remodeler with links to mitochondrial dynamics, we explored how its depletion impacts ROS levels and genomic stability. We investigated ROS levels and observed that the depletion of HELLS and MIEF1 led to decreased ROS (Fig. 7A and Supplementary Fig. S9A) and reduced the length of DNA damage tails (Fig. 7B), indicating that HELLS depletion decreases the level of DNA damage and enhances genome stability. Additionally, HELLS loss reduced levels of phosphorylated H2AX (γH2AX), a typical DNA damage marker (Fig. 7C), and induced less sensitivity to changes in γH2AX upon cisplatin treatment (Supplementary Fig. S9B). In contrast, HELLS overexpression in HepG2 and non-tumorigenic MIHA cells increased ROS production and DNA damage (Fig. 7D, E and Supplementary Fig. S9C, D). These findings suggest that HELLS contributes to genome instability by regulating ROS levels and DNA damage sensitivity (Fig. 7F).

### MIEF1 is a key executor in the HELLS-MIEF1 axis

**Fig. 8:**
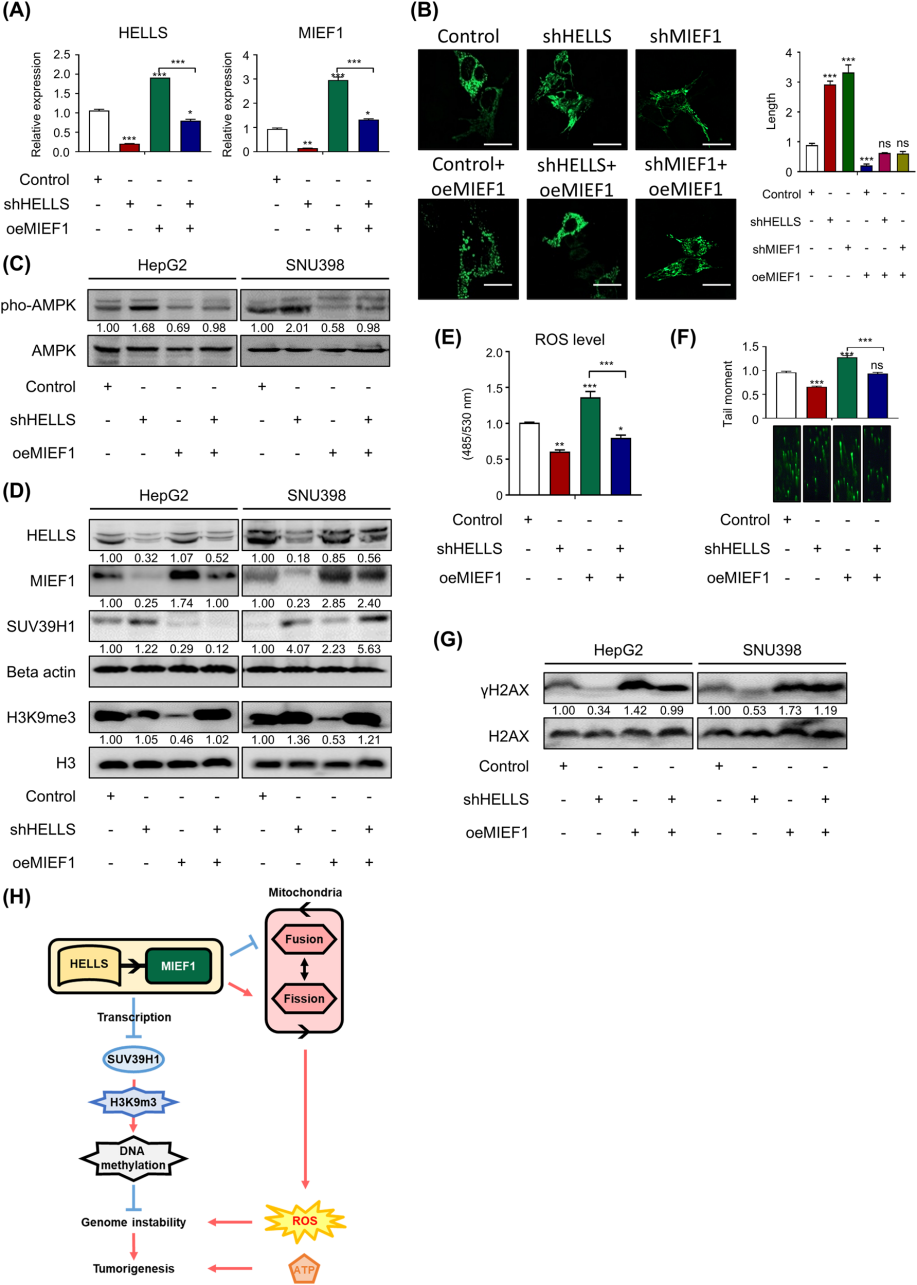
MIEF1 overexpression reverses the effects induced by HELLS-MIEF1 axis suppression. **A** The relative mRNA levels of HELLS and MIEF1 in HepG2 cells treated with Control, shHELLS, oeMIEF1, and shHELLS+oeMIEF1 were presented as mean ± S.E.M. (*p < 0.05, **p < 0.01, ***p < 0.001, *n* = 3). **B** Representative confocal images of mitochondria. HepG2 cells treated with Control, shHELLS, shMIEF1, oeMIEF1, shHELLS+oeMIEF1, and shMIEF1+oeMIEF1 were transfected with mt-ro2GFP. The scale bar represents 20 μm. Quantification of mitochondrial length was measured (mean ± S.E.M., ns non-significant, ***P < 0.001, *n* = 3). **C** Protein levels of pho-AMPK and AMPK in HepG2 and SNU398 cells treated with Control, shHELLS, oeMIEF1, and shHELLS+oeMIEF1 were measured using western blots. **D** Protein levels of HELLS, MIEF1, SUV39H1, Beta actin, H3K9me3, and Histone H3 in HepG2 and SNU398 cells treated with Control, shHELLS, oeMIEF1, and shHELLS+oeMIEF1 were measured using western blots. **E** ROS levels in HepG2 cells treated with Control, shHELLS, oeMIEF1, and shHELLS+oeMIEF1. (mean ± S.E.M., *p < 0.05, **p < 0.01, ***p < 0.001, *n* = 3). **F** Representative comet images stained with a green DNA staining solution and a bar graph showing the quantification of a tail moment in HepG2 cells treated with Control, shHELLS, oeMIEF1, and shHELLS+oeMIEF1 (mean ± S.E.M., ns non-significant, ***p < 0.001, *n* = 3). **G** Protein levels of γH2AX and H2AX in HepG2 and SNU398 cells treated with Control, shHELLS, oeMIEF1, and shHELLS+oeMIEF1 were measured using western blots. **H** The graphical summary illustrates the proposed model based on the overall findings of this study.

To investigate the compensatory role of MIEF1 in the context of HELLS-MIEF1 axis reduction, we overexpressed MIEF1 in HepG2 cells and examined its effects on the resulting phenotypic changes. Overexpression of MIEF1 in HepG2 cells restored the mitochondrial morphology from the elongated fusion state induced by HELLS or MIEF1 knockdown and further recovered mitochondrial function and the energy level (Fig. 8A, B). This restoration was confirmed by the observed reduction in phoAMPK levels upon MIEF1 overexpression (Fig. 8C). Furthermore, MIEF1 overexpression reversed the increase in SUV39H1 and H3K9me3 levels resulting from HELLS knockdown (Fig. 8D). Notably, reductions in ROS levels, shortened DNA damage tails, and decreased γH2AX observed upon HELLS knockdown were all rescued by MIEF1 overexpression (Fig. 8E–G). These findings demonstrate that MIEF1 overexpression can restore mitochondrial morphology, function, and genome stability by compensating for the effects of HELLS-MIEF1 axis reduction. To further investigate the relationship between mitochondrial dynamics and genomic stability under the HELLS-MIEF1 axis, we treated HCC cells with BGP15, a pharmacological inducer of mitochondrial fusion [29]. BGP15 treatment mimicked the fused mitochondrial morphology observed in HELLS knockdown (Supplementary Fig. S10A). BGP15 treatment increased H3K9me3 levels accompanied by reduced HELLS and MIEF1 expression levels (Supplementary Fig. S10B and C). Cells also exhibited reduced ROS levels and decreased DNA damage (Supplementary Fig. S10D–F). These results highlight the role of mitochondrial fusion in maintaining genomic stability and support the importance of the HELLS-MIEF1 axis in HCC.

## Discussion

In this study, we propose that the HELLS–MIEF1 axis is aberrantly activated and plays an oncogenic role in liver cancer. Our comprehensive analyses demonstrate that HELLS regulates mitochondrial dynamics and cellular metabolism through the transcriptional control of MIEF1. The reduction of the HELLS-MIEF1 axis leads to significant cellular energy deprivation and induces senescence, as evidenced by decreased ATP due to mitochondrial hyperfusion. This depletion results in reduced ROS production and DNA damage, contributing to genome stabilization, which is further supported by SUV39H1-mediated increases in H3K9me3 and elevated levels of DNA methylation. These results underscore the critical role of the HELLS–MIEF1 axis in liver cancer and provide valuable insights into the communication between nuclear and mitochondrial processes (Fig. 8H).

Common oncogenic signaling converges with changes to mitochondrial morphology, which are strongly associated with metabolism and complex signals involved in various aspects of tumor formation [21]. Most cancers, particularly liver cancer, tend to favor fragmented mitochondria [29–31]. However, the detailed interplay between mitochondrial dynamics and oncogenesis has not been thoroughly studied. Our study identifies MIEF1 as a new oncogene candidate, directly targeted by HELLS and acting as a fission protein (Figs. 1 and 2). The HELLS–MIEF1 axis emerges as a key regulator of metabolism, contributing to tumor growth by regulating energy production (Fig. 3). Pioneer studies have shown that HELLS acts as a tumor suppressor and influences cellular metabolism, such as lactate production [22] or the regulation of tricarboxylic acid (TCA) cycle intermediates through its control of fumarate hydratase expression [32]. However, a comprehensive understanding of the precise mechanism of its phenotypes remains elusive. Our study is the first to demonstrate an association between HELLS and a mitochondrial dynamics protein, establishing a novel link between nuclear chromatin remodeling and mitochondrial function. By manipulating the HELLS-MIEF1 axis, we induced a state of mitochondrial hyperfusion, leading to dysfunctional mitochondrial hyperfusion and eliciting an anti-cancer effect. This finding not only expands our understanding of the HELLS-MIEF1 axis but also suggests potential therapeutic strategies targeting mitochondrial dynamics in liver cancer.

Several studies have highlighted the association between HELLS and histone modifications [27, 32, 33]. Specifically, the absence of HELLS expression led to elevated H3K4me2 and H3K4me3 levels at hypomethylated pericentromeric DNA and other repetitive sequences in mice [32], as well as increased H3K27me3 and H3K4me1 levels at CpG hypomethylated sites in murine embryonic fibroblasts [27, 33]. However, studies focusing on HELLS and H3K9me3 are limited. Notably, HELLS mutant oocytes did not exhibit significant changes in H3K9me3 levels in one study [34], and the depletion of HELLS in 1BR-hTert cells barely affected overall H3K9me3 levels in another study [35]. In our study, HELLS knockdown increased H3K9me3 accompanied by upregulation of SUV39H1, particularly in genomic hotspots, stabilizing the genome along with other heterochromatin markers, such as H3K20me3 and H1, and DNA methylation (Figs. 4–6). Elevated H3K9me3 levels have two implications in cancer [36]: an increase in H3K9me3 levels in specific promoters is strongly associated with the suppression of tumor suppressor gene expression [37], and therefore inhibition of SUV39H1 or SETDB1 has been proposed as a promising therapeutic strategy in HCC [36, 38]. In comparison, decreased H3K9me3 indicates genomic instability and leads to proto-oncogenes [39–42]. Few studies have focused on genome-wide reduced H3K9me3 in HCC. Our findings provide initial insights into this phenomenon, and further research is required to resolve the H3K9me3 paradox in cancer.

Most studies using HELLS-knockout mice or HELLS-depleted cells have demonstrated that the loss of HELLS expression results in global DNA hypomethylation, albeit with subtle differences in the extent and specific sites affected. These studies typically report decreased DNA methylation across various genomic regions, including pericentromeric DNA and other repetitive sequences. Contrary to previous findings, our study showed that HELLS knockdown in liver cancer cell lines resulted in hypermethylation in 89% of the probes analyzed. This hypermethylation was further confirmed by dot-blot analysis, which indicated a concurrent reduction in 5hmC levels. Interestingly, most hypermethylated regions identified were closed regions rather than CpG islands (Fig. 6). The discrepancy in these results could be attributed to the different cell types used or variations in the coverage and sensitivity of the methodologies applied.

Understanding the intricate relationship between ROS generation and mitochondrial shape and function is complex. Typically, mitochondrial fusion is associated with high levels of energy and ROS production [43, 44]. Interestingly, suppression of the HELLS-MIEF1 axis led to an over-fused state with reduced levels of energy and ROS (Figs. 7 and 8), indicative of dysfunctional mitochondria. This hyperfused state suggests that an imbalance in mitochondrial dynamics can impair mitochondrial function and energy production. Additionally, the reduction in ROS levels observed under these conditions contributes to decreased oxidative stress, which leads to a globally stabilized genome. This stabilization is evidenced by increased H3K9me3 and DNA methylation, which collectively cause cancer cells to age prematurely. In this regard, previous studies have demonstrated an association between HELLS and DNA damage repair (DDR) pathways, with evidence suggesting its ability to modulate γH2AX levels depending on the cellular context [10, 35, 45]. In our findings, decreased DNA damage and γH2AX levels were observed upon HELLS knockdown. These findings underscore the critical relationship among ROS generation, mitochondrial dynamics, and genome instability, highlighting the role of the HELLS-MIEF1 axis on cellular function and integrity.

In summary, we demonstrated that the HELLS-MIEF1 axis is crucial in liver cancer by modulating mitochondrial dynamics, cellular metabolism, and genomic stability. Disruption of this axis leads to significant metabolic and epigenetic consequences, particularly affecting ROS production and mitochondrial function. This study highlights the intricate crosstalk between the nucleus and mitochondria facilitated by the HELLS-MIEF1 axis, underscoring its role in both mitochondrial function and epigenetic regulation. Targeting this axis presents a dual-target strategy, offering potential for innovative cancer therapies that address both metabolic dysregulation and genomic instability, ultimately enhancing the effectiveness of existing treatments.

## Supplementary information

supplementary information

supplementary file_Original western blots

## Data Availability

The accession number for gene expression data reported in this study is NCBI GEO: GSE212554. The accession number for methylation data reported in this study is NCBI GEO: GSE212556. The accession number for H3K9me3 ChIP seq data reported in this study is NCBI GEO: GSE212429.
