# Supplementary material for: HELLS controls mitochondrial dynamics and genome stability in liver cancer by collusion with MIEF1
Source: Cell Death Dis. 2025 Apr 2;16(1):239. doi: 10.1038/s41419-025-07589-x (PMC11965466; doi:10.1038/s41419-025-07589-x)
Supplement: Supplementary file 1 — supplementary information [file 41419_2025_7589_MOESM1_ESM.docx]

**HELLS controls mitochondrial dynamics and genome stability in liver cancer by collusion with MIEF1**

Authors: Sung Kyung Choi, Jihye Park, Sang Yun Ha, Myoung Jun Kim^,^ Seor I Ahn, Jeongah Kim, Woong Sun, Yeong Min Park, Suk Woo Nam, Jeung-Whan Han, Keunsoo Kang, and Jueng Soo You

**Table of contents**

Supplementary Methods……………………………………………………………………………………………………………………2

Supplementary Figures ……………………………………………………………………………………………………………………12

Supplementary Tables………………………………………………………………………………………………………………………28

**Supplementary Methods**

**Transient transfections**

Cells with ectopic expression of HELLS or MIEF1 were produced by transfecting the respective expression vectors. HepG2, Huh7, SNU398, and MIHA cells were seeded in six-well plates and transiently transfected with either the HELLS overexpression vector (ORIGENE, RC212231) or the MIEF1 overexpression vector (ORIGENE, RC203568) using Lipofectamine 2000 reagent (Thermo Fisher Scientific). 16 hours after transfection, the Opti-MEM medium was replaced with growth media.

**RNA extraction and reverse transcription PCR**

Cells were collected, and total RNA was extracted using TRIzol reagent and digested with DNase I. cDNA synthesis from total RNA was performed using the High-Capacity cDNA Reverse Transcriptase Kit (Applied Biosystems) following the manufacturer's instructions. Amplification of cDNA was performed on a LightCycler 480II (Roche) using the LightCycler 480 SYBR Green I Master (Roche), according to the recommended conditions. The average Ct value for each gene was determined from triplicate reactions and normalized to the mRNA levels of GAPDH. cDNAs were amplified using gene-specific primers listed in Supplementary Table 1.

**Western blot analysis**

Cells were lysed with RIPA buffer and briefly sonicated. Cell lysates were boiled in Laemmli sample buffer, and 30 μg of each protein sample was subjected to SDS-PAGE. Protein concentration was measured using the Bradford protein assay. Samples were separated on 8–15% SDS-PAGE and transferred to nitrocellulose membranes. After blocking for 60 minutes with 10% milk in TBS-T and washing three times for 5 minutes each, membranes were incubated overnight with primary antibodies. After three washes for 5 minutes each, membranes were incubated for 60 minutes at room temperature with secondary antibodies. Antibodies are listed in Supplementary Table 3. The intensity of protein bands was quantified using ImageJ.

**Chromatin immunoprecipitation assay (ChIP)**

ChIP assays were performed according to the instructions from Upstate Biotechnology. Protein was cross-linked to DNA with 1% formaldehyde. For each assay, 50 μg of DNA, sheared by sonication (with DNA fragment sizes ranging from 200 to 500 bp), was precleared with protein A magnetic beads (Upstate Biotechnology, #16-661), and then 50 μg of DNA was precipitated using antibodies against HELLS and H3K4me3. After immunoprecipitation overnight at 4°C, the recovered chromatin fragments were subjected to real-time PCR. IgG control experiments were performed for all ChIPs, and the results were incorporated into the IP/Input (1%) by presenting the results as (IP-IgG)/(Input-IgG). ChIP primers and antibodies are listed in Supplementary Tables 2 and 3.

**ChIP-sequencing**

The construction of the library was performed using the NEBNext Ultra DNA Library Prep Kit. Briefly, the sheared DNA was ligated with adaptors. After purification, a PCR reaction was performed with adaptor-ligated DNA and index primers for multiplex sequencing. The library was purified using magnetic beads to remove all reaction components. The size of the library was assessed using an Agilent 2100 Bioanalyzer. High-throughput sequencing was performed as paired-end 150 sequencing using a NovaSeq 6000. This data set was obtained from the National Center for Biotechnology Information (NCBI) Gene Expression Omnibus (GEO) database (accession no. GSE212429).

**ChIP-seq data analysis**

Low-quality and/or adapter-contaminated portions of sequenced reads were trimmed using Trim Galore (version 0.6.6) with Cutadapt (version 3.4). Trimmed reads were then aligned to the reference human genome (GRCh37) using Bowtie2 (version 2.4.5) with the following parameters: "--local --very-sensitive-local --no-mixed --no-discordant," and gene annotation information from GENCODE release 19. Aligned reads showing low quality (mapping quality less than 10) were further removed using Samtools (version 1.10). Additionally, Sambamba (version 0.8.2) was used to remove duplicated reads with the following parameters: "markdup -r."

To identify reliable peaks, two different approaches were sequentially used. First, initial peaks were identified using MACS3 (version 3.0.0a7) with the following parameters: "-B -g hs --broad." Then, potential enrichment noises were deducted using MACS3's bdgcmp function, which compared two signal tracks (bedGraph files) generated by the above analysis. Finally, reliable peaks were identified by analyzing the bdgcmp output with the bdgbroadcall function of MACS3, using the following parameters: "-G 2000 -c 2 -C 0.5."

To visualize the enrichment of H3K9me3 in the identified peaks, bigwig files were generated using the bedGraphToBigWig tool with the mapped read files. Deeptools (version 3.5.1) was then used to draw heatmaps with default parameters. This data set was obtained from the NCBI Gene Expression Omnibus (GEO) database (accession no. GSE212429).

**Gene ontology analysis**

DAVID, Metascape, and EnrichR were used to infer the biological functions of genes associated with peaks. Default parameters were used for all analyses.

**Microarray**

Labeled cRNA was prepared from 1–5 μg of total RNA using Agilent's Quick Amp Labeling Kit. Following fragmentation, 1.65 μg of cRNA was hybridized to the Agilent expression microarray according to the protocols provided by the manufacturer. Arrays were scanned using the Agilent Technologies G4900DA SG12494263. Array data export, processing, and analysis were performed using Agilent Feature Extraction v11.0.1.1. This data set was obtained from the NCBI Gene Expression Omnibus (GEO) database (accession no. GSE212554).

**EPIC**

Genome-wide DNA methylation profiling was performed using the Illumina Infinium Methylation EPIC Beadchip array (Illumina, San Diego, CA, USA) following the manufacturer's instructions. Genomic DNA was bisulfite converted using the EZ DNA Methylation Kit (Zymo Research, Irvine, CA, USA). Bisulfite-treated samples were then amplified, fragmented, purified, and hybridized onto the EPIC Beadchip according to the manufacturer's standard protocol. The arrays were washed and scanned using the Illumina HiScan System. This data set was obtained from the NCBI Gene Expression Omnibus (GEO) database (accession no. GSE212556).

**International Cancer Genome Consortium data analysis**

RNA expressions of HELLS and MIEF1 were analyzed using RNA-seq-based gene expression data from the International Cancer Genome Consortium (ICGC) liver hepatocellular carcinoma (HCC) project. RNA-seq data were analyzed by first replacing all RSEM values equal to zero with the smallest nonzero RSEM value, followed by a log2 transformation.

**Cell cycle analysis**

Cells were collected by trypsinization and cell-cycle assays were performed using the CycleTEST Plus DNA Reagent Kit (BD Biosciences). The cell cycle profiles were analyzed using a BD Accuri™ C6 (BD Biosciences).

**Soft agar colony-forming assay**

The assay was performed in six-well plates. A bottom layer of agar (0.5%) with enriched DMEM media (10% FBS) was poured first. After the bottom agar solidified, cells (1.0 × 10⁴) were seeded in top agar (0.3%) with enriched DMEM supplemented with 10% FBS and incubated at 37°C for 3 weeks. The culture medium was changed once or twice weekly. Colonies were visualized by staining for 1 hour with 0.005% crystal violet.

**Wound healing assay**

Cells were grown to confluence in six-well plates. After overnight starvation in serum-free medium, cell monolayers were scraped with a sterile micropipette tip. Initial gap widths (0 hour) and residual gap widths at 8, 24, 48, and 72 hours after wounding were determined from photomicrographs.

**Proliferation**

Cells were plated at a density of 5 × 10⁴ cells/well in six-well plates. Viable cell numbers were measured daily using Trypan blue exclusion and counting with a microscope.

**Immunocytochemistry**

Cells seeded in 12-well plates containing a glass coverslip were washed with 1X PBS and then fixed with 4% neutral buffered formaldehyde solution for 30 minutes at room temperature. Cells were treated with 0.1% Triton X-100 in PBS for 5 minutes at room temperature. After being blocked with 1% goat serum/PBS for 30 minutes at room temperature, cells were incubated with primary antibody (in 1% goat serum/PBS) for 30 minutes at room temperature. After washing three times with PBS, the FITC-tagged secondary antibody (in 1% BSA/PBS) was added to the cells and incubated at room temperature for 30 minutes. Nuclei were stained with TOPRO (Invitrogen, T3605). The slides were washed in PBS and mounted with a mounting medium. The results were visualized using an Olympus confocal laser scanning microscope. Antibodies are listed in Supplementary Table 3.

**PEG (polyethylene glycol) cellular fusion assay**

Cells were transiently transfected with either MitoTracker Green (Invitrogen, M7514) or MitoTracker Deep Red (Invitrogen, M22426). At 8 hours after transfection, cells were co-plated (ratio 1:1) on coverslips and co-cultivated for 16 hours. The fusion of co-cultured cells was induced by a 90-second treatment with a pre-warmed solution of 50% PEG 3350 in PBS, followed by washing with pre-warmed PBS. After an additional 5 hours of co-cultivation in cell culture medium, cells were fixed with 4% formaldehyde in PBS. After two washes with PBS, coverslips were mounted. To inhibit de novo synthesis of fluorescent proteins, cells were incubated with the protein synthesis inhibitor cycloheximide (40 μg/ml) for 30 minutes before PEG treatment, and cycloheximide was subsequently added to all solutions and tissue culture media until the cells were fixed. The results were visualized using an Olympus confocal laser scanning microscope. The percentage of mitochondrial fusion was determined by the overlap rate between green and red mitochondrially targeted fluorescent proteins expressed in one cell hybrid.

**Immunohistochemistry**

IHC was performed on tissue microarray (TMA) blocks consisting of 2 mm cores obtained from 238 HCCs and two normal liver tissues after approval by the institutional review board of Samsung Medical Center. The sections were incubated with anti-HELLS antibody (1:300 dilution; Santa Cruz; # sc-46665) and anti-MIEF1 antibody (1:150 dilution; Proteintech, # 20164-1-AP) overnight in a cold room after antigen retrieval with TE buffer (10 mmol/L Tris-1 mmol/L EDTA, pH 9.2). Sections were then incubated with an anti-mouse/rabbit IgG antibody (Thermo Fisher Scientific) for 20 minutes at room temperature. Antigen–antibody chromogenic reactions were developed for 30 minutes and detected using the REAL EnVision detection system K5007. IHC staining was analyzed by a liver pathologist (SYH) without prior knowledge of clinicopathological results. High expression of HELLS was defined as nuclear staining in more than 10% of tumor cells. High expression of MIEF1 was defined as nuclear and cytoplasmic staining in more than 10% of tumor cells and further divided into two subgroups according to staining intensity: (+) weak staining and (++) moderate to strong staining.

**Measurement of mitochondrial morphology and structure**

To observe the mitochondrial morphology, cells were co-transfected with Mt-ro2GFP (Addgene, #82408). After 48 hrs, cells were fixed with 4% paraformaldehyde for 15 min at room temperature (RT) and washed with PBS three times. Coverslips were mounted and imaged by a Zeiss LSM 800 confocal microscopy with Airyscan. Images were acquired by a Zeiss LSM 800 confocal microscopy and processed using image J program. To analyze mitochondria morphology, mitochondria were marked with GFP through the transfection of Mt-ro2GFP. The mitochondrial area and number were measured by 'analyze particle' from processed binary images.

**Transmission electron microscopy.**

Cells were collected for examination. The samples were fixed using a fixative solution composed of 2.5% glutaraldehyde in 0.1M phosphate buffer (PB) at pH 7.4. After primary fixation, the samples were washed in phosphate buffer (PB) and then post-fixed in 1% osmium tetroxide (OsO₄) in 0.1M phosphate buffer. The samples were gradually dehydrated through a series of ethanol solutions with increasing concentrations (30%, 50%, 70%, 80%, 90%, 95%, and 100%) to remove water from the tissue. Following dehydration, the samples were infiltrated with a mixture of ethanol and Epon 812 resin in ratios of 3:1, 1:1, and 1:3, respectively. The samples were then embedded in 100% Epon 812 resin and left overnight. Fresh pure Epon 812 was added and incubated for 6 hours. The resin was allowed to polymerize by incubating the samples in a 60°C oven. Using an ultramicrotome (EM UC7, Leica, Germany), ultrathin sections of 100 nm thickness were cut from the embedded samples. These sections were collected on copper grids. The sections were stained with 1% uranyl acetate followed by lead citrate. These stains enhanced contrast by binding to different cellular structures, making the mitochondria more visible under the electron microscope. Finally, the stained sections were observed using a transmission electron microscope (TEM) (HT7800, Hitachi, Japan) at an accelerating voltage of 80-100 kV to visualize the mitochondria and other cellular structures.

**Assessment of mitochondrial membrane potential**

To label the mitochondria and observe the mitochondrial membrane potential, cells were co-transfected with Mt-ro2GFP. After 48 hrs, cells were stained with 50 nM Tetramethylrhodamine, methyl ester (TMRM) (Invitrogen, T668) in phenol red-free DMEM containing 10% FBS at 37°C with 5% CO2 for 15 min and were examined using a Zeiss LSM 800 confocal microscopy with Airyscan. The fluorescence intensity of TMRM were measured by using ImageJ software. The three regions of interests (ROIs) from mitochondrial regions per each cell were

**In vivo tumor growth experiment**

BALB/c nude mice (male, 6–7 weeks old, 25 g) were purchased from Orient Bio and maintained under pathogen-free conditions. Huh7 cells (5 × 10⁶ cells per 0.1 mL Hank's Balanced Salt Solution) were injected subcutaneously into the right groin. The mice were monitored daily, and tumor sizes were measured every 2 to 3 days using a digital caliper. Tumor volumes were calculated using the formula volume = π/6 (length × width²). Randomization and blinding were not performed because all animals were subjected to identical experimental conditions without grouping or treatment variability. The animal study was reviewed and approved by the Institutional Animal Care and Use Committee of Konkuk University.

**Extracellular acidification rate (ECAR)**

A Glycolysis Stress Test (103020-100, Agilent, Santa Clara, CA) was performed using the Seahorse XFe96 Extracellular Flux Analyzer (Agilent, Santa Clara, CA). In total, 6 × 10⁴ cells were plated into wells of an XF96 Cell Culture Microplate (102416-100, Agilent, Santa Clara, CA) and incubated overnight. Plates were equilibrated in unbuffered XF assay medium supplemented with 2 mM glutamine without CO₂ for 1 hour. Extracellular acidification rates (ECAR) were assayed by the serial addition of glucose (10 mM), oligomycin (1 μM), and 2-deoxy-glucose (2-DG, 50 mM) to determine glycolysis (ECAR in response to glucose − ECAR before glucose injection), glycolytic capacity (ECAR in response to oligomycin − ECAR before glucose injection), glycolytic reserve (glycolytic capacity − glycolysis), and non-glycolytic acidification (ECAR before glucose injection). Each plotted value is the mean of at least triplicate wells and normalized to baseline ECAR and total protein levels. Data are presented as mean ± S.D., and significance is calculated by two-way ANOVA with Tukey's multiple comparison test.

**Oxygen consumption rate (OCR)**

A Cell Mito Stress Test (103015-100, Agilent, Santa Clara, CA) was measured using the Seahorse XFe96 Extracellular Flux Analyzer (Agilent, Santa Clara, CA). In total, 6 × 10⁴ cells were plated into each well of an XF96 Cell Culture Microplate (102416-100, Agilent, Santa Clara, CA) and incubated overnight. The plates were equilibrated in unbuffered XF assay medium supplemented with 2 mM glutamine without CO₂ for 1 hour. Oxygen consumption rates (OCR) were assessed by the serial addition of oligomycin (1 μM), carbonyl cyanide 4‐(trifluoromethoxy) phenylhydrazone (FCCP, 1 μM), and rotenone/antimycin A (Rot/AA, 1 μM) to determine basal respiration (OCR before oligomycin injection − OCR in response to Rot/AA), ATP production (OCR before oligomycin injection − OCR in response to oligomycin), maximal respiration (OCR in response to FCCP − OCR in response to Rot/AA), spare capacity (maximal respiration − basal respiration), and proton leak (OCR in response to oligomycin − OCR in response to Rot/AA). Each plotted value is the mean of at least three triplicate wells and normalized to baseline OCR and total protein levels. Data are presented as mean ± S.D., and significance is calculated by two-way ANOVA with Tukey's multiple comparison test.

**Measurement of intracellular ROS generation**

Cells were distributed into a 24-well culture plate and incubated for 24 hours in a CO₂ incubator. Cells were washed with HBSS (Sigma, H6648) and incubated with 20 μM carboxy-H2DCF-DA (Molecular Probes #C-400) in HBSS in a CO₂ incubator at 37°C for 10 minutes. Cells were washed with HBSS to remove carboxy-H2DCF-DA, replaced with 1 mL of HBSS, and incubated for 120 minutes. Cells were washed with cold HBSS twice and then incubated with 0.5% Triton X-100 (diluted in PBS) for 5 minutes. Fluorescence was measured using a fluorescence multi-well plate reader with excitation and emission wavelengths of 485 nm and 530 nm, respectively.

**Comet assay**

DNA damage was measured using the OxiSelect™ Comet Assay Kit (3-Well Slides) according to the manufacturer's protocol (Cell Biolabs, STA-351).

**Senescence-associated beta-galactosidase (SA-β-gal) detection assay**

Cells were washed with PBS, followed by fixation at room temperature for 5 minutes. Subsequently, cells were incubated in a staining solution for senescence-associated β-galactosidase (SA-β-gal) with a pH of 6.0 overnight at 37°C. The fixed and stained cells were rinsed with methanol, air-dried, and then subjected to washing and observation using a bright-field microscope.

**Statistical analysis**

Statistical tests were chosen based on the experimental design and data characteristics. Parametric tests such as t-tests and ANOVA were used for normally distributed data.

**Supplementary Figures**

**
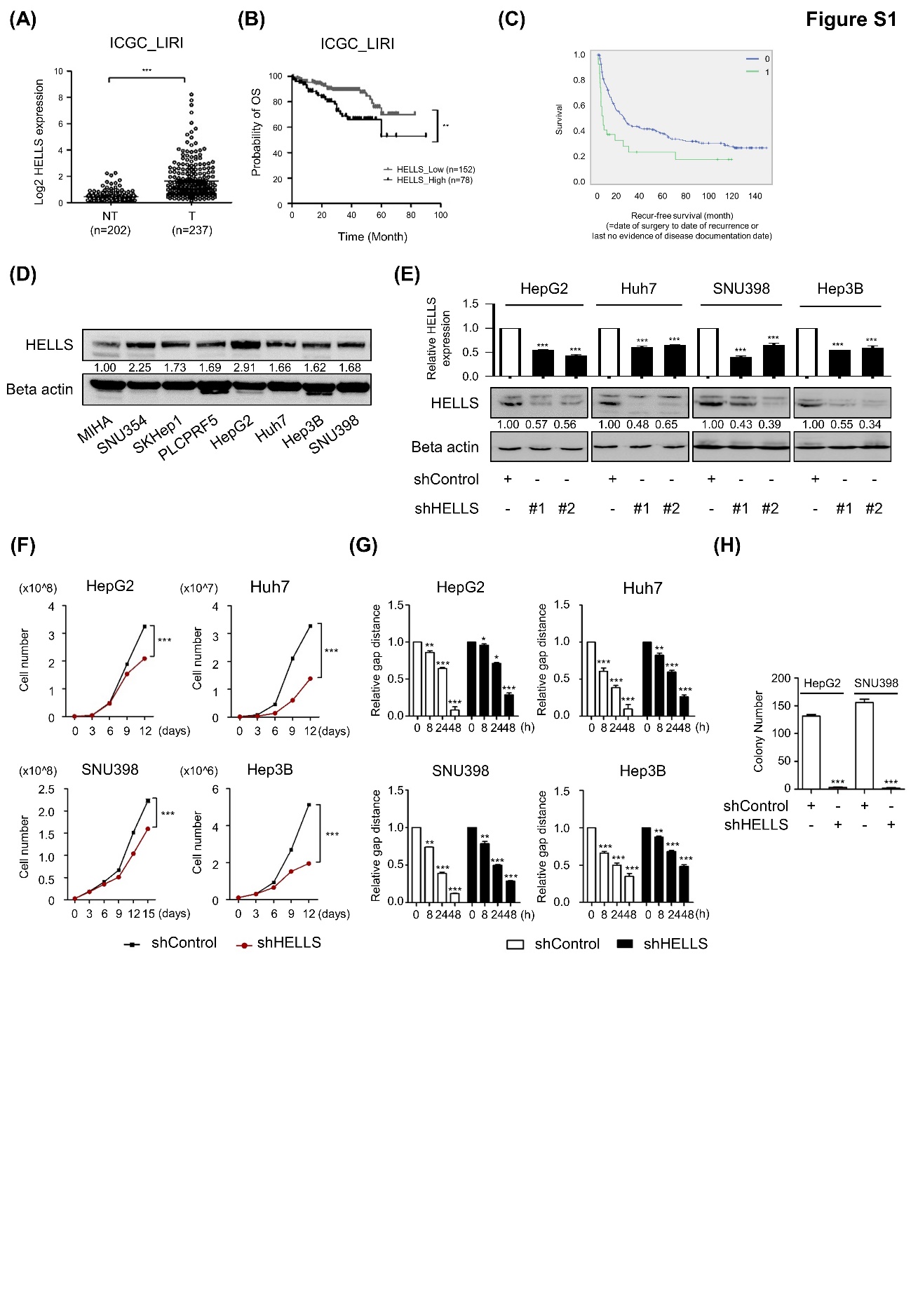
**

**Figure S1. HELLS is overexpressed and acts as an oncogene in liver cancer.**

1. HELLS expression in non-tumor (NT) and tumor (T) tissues presented as mean ± S.D. (***p < 0.001). Horizontal lines represent the median.
2. Kaplan–Meier survival analysis based on HELLS levels. The p-value was calculated using the log-rank test (**p< 0.01).
3. Recurrence-free survival time dependent on HELLS expression (0: HELLS negative, 1: HELLS positive).
4. Protein levels of HELLS and Beta actin in normal liver cell line and liver cancer cell lines.
5. HELLS expression levels upon HELLS depletion. mRNA and protein levels of HELLS and Beta actin in HepG2, Huh7, SNU398, and Hep3B cells measured using qRT-PCR and Western blots.
6. Growth rates upon HELLS loss. Cell numbers in HepG2, Huh7, SNU398, and Hep3B cells presented as mean ± S.E.M. (***p < 0.001, n=3).
7. Cell migration rates measured by wound healing assay in HepG2, Huh7, SNU398, and Hep3B cells upon HELLS loss (mean ± S.E.M., *p < 0.05, **p < 0.01, ***p < 0.001, n=3).
8. Colony formation measured by soft agar assay in HepG2 and SNU398 cells upon HELLS loss (mean ± S.E.M., ***p < 0.001, n=3).

**
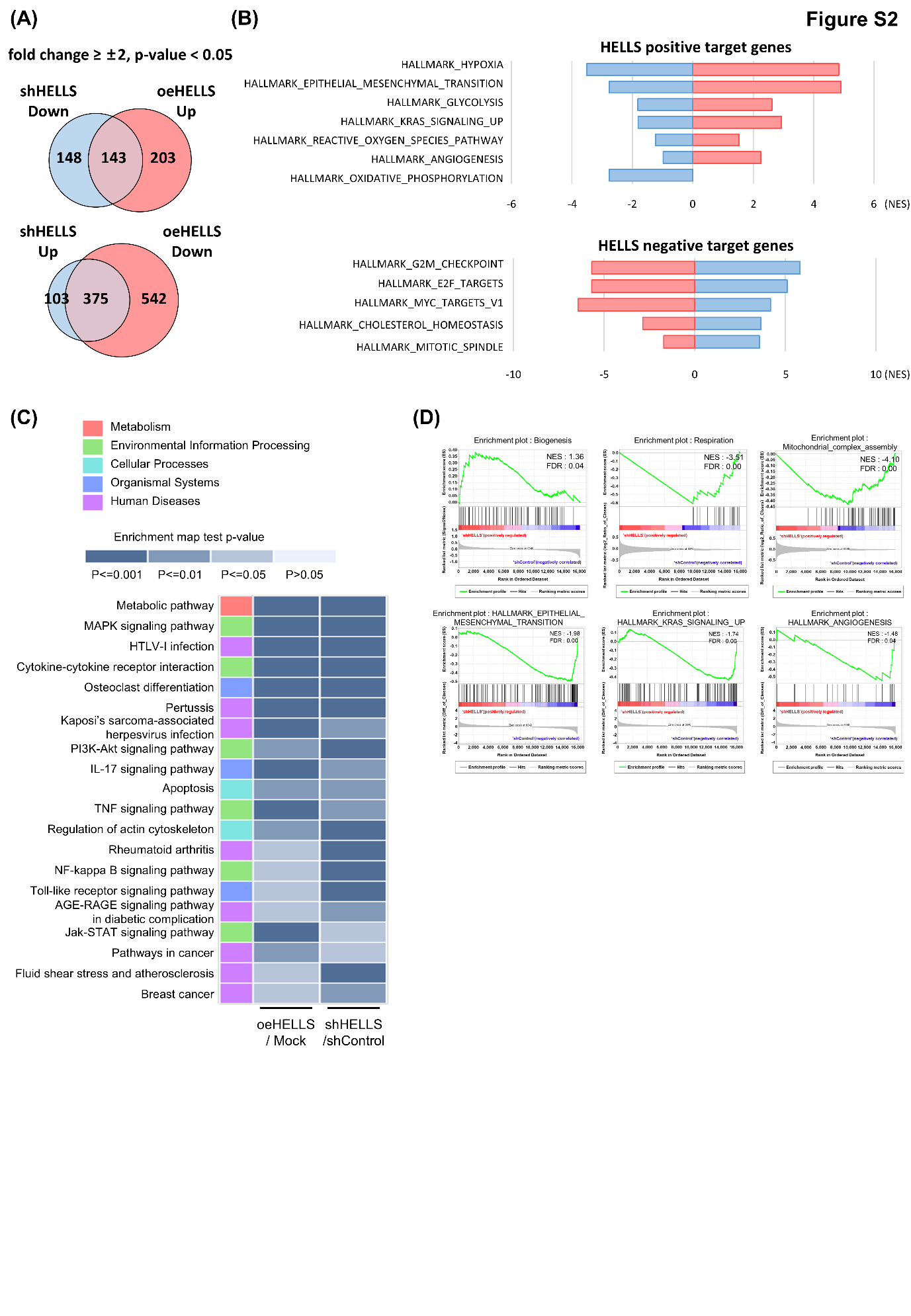
**

**Figure S2. HELLS plays a role in oncogenic and metabolic pathways in liver cancer.**

1. Number of DEGs downregulated by HELLS knockdown and upregulated by HELLS overexpression, and number of DEGs upregulated by HELLS knockdown and downregulated by HELLS overexpression (fold change ≥ ±2, p-value < 0.05).
2. Hallmark analysis of HELLS positive target genes (143) and HELLS negative target genes (375).
3. KEGG pathway analysis of differentially expressed genes following HELLS overexpression and knockdown.
4. Gene set enrichment analysis (GSEA) of HELLS depletion. NES (Normalized Enrichment Score), FDR (FDR q-value).

**
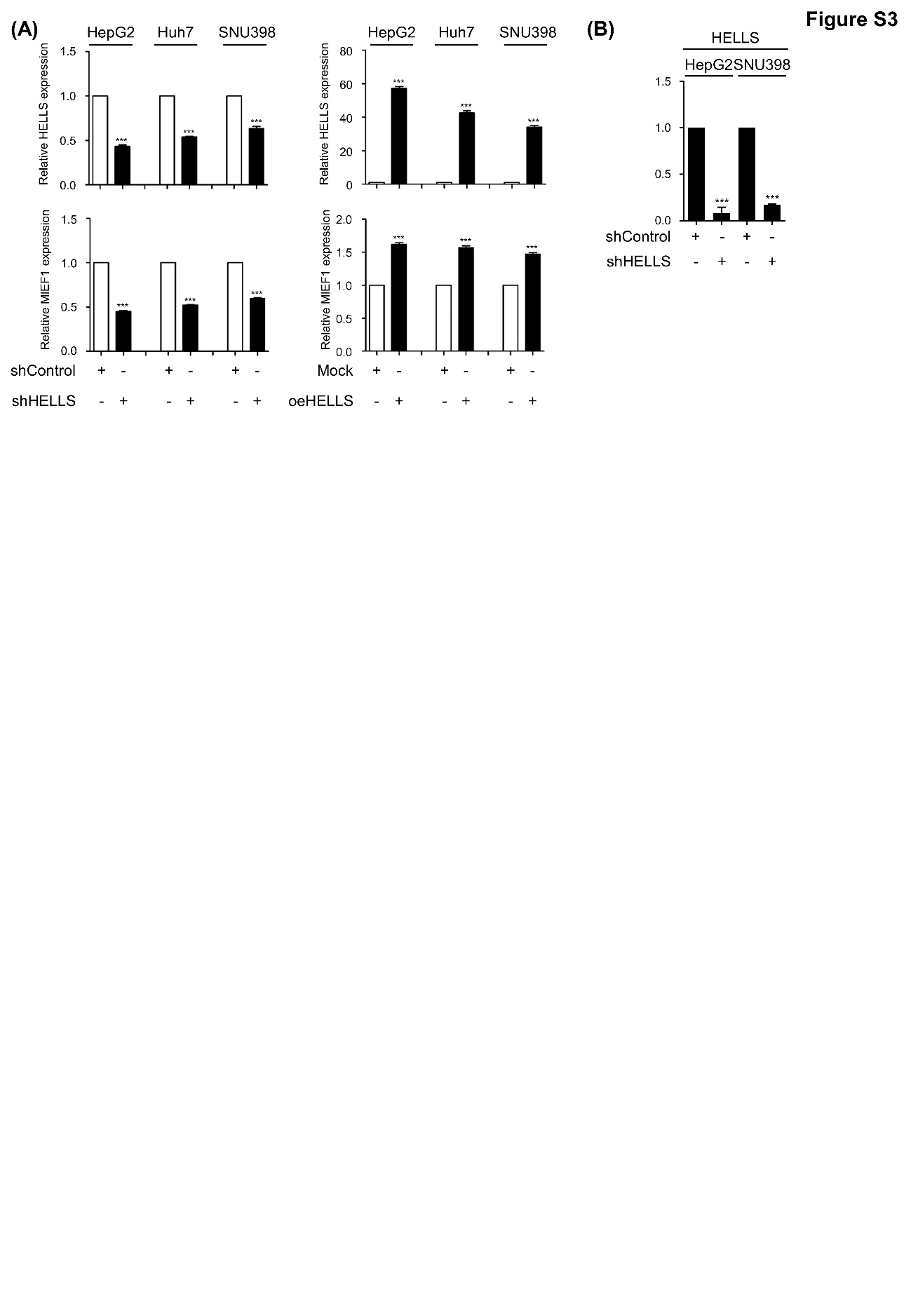
**

**Figure S3. HELLS directly regulates the transcription of MIEF1 in liver cancer cells.**

1. Expression levels of HELLS and MIEF1 in HepG2, Huh7, and SNU398 cells following HELLS knockdown or overexpression, as determined by qRT-PCR (mean ± S.E.M., ***p < 0.001, n=3).
2. HELLS binding at the MIEF1 promoter. The enrichment of HELLS at the MIEF1 promoter in HepG2 and SNU398 cells was presented using ChIP-qPCR (mean ± S.E.M., ***p < 0.001, n=3).

**
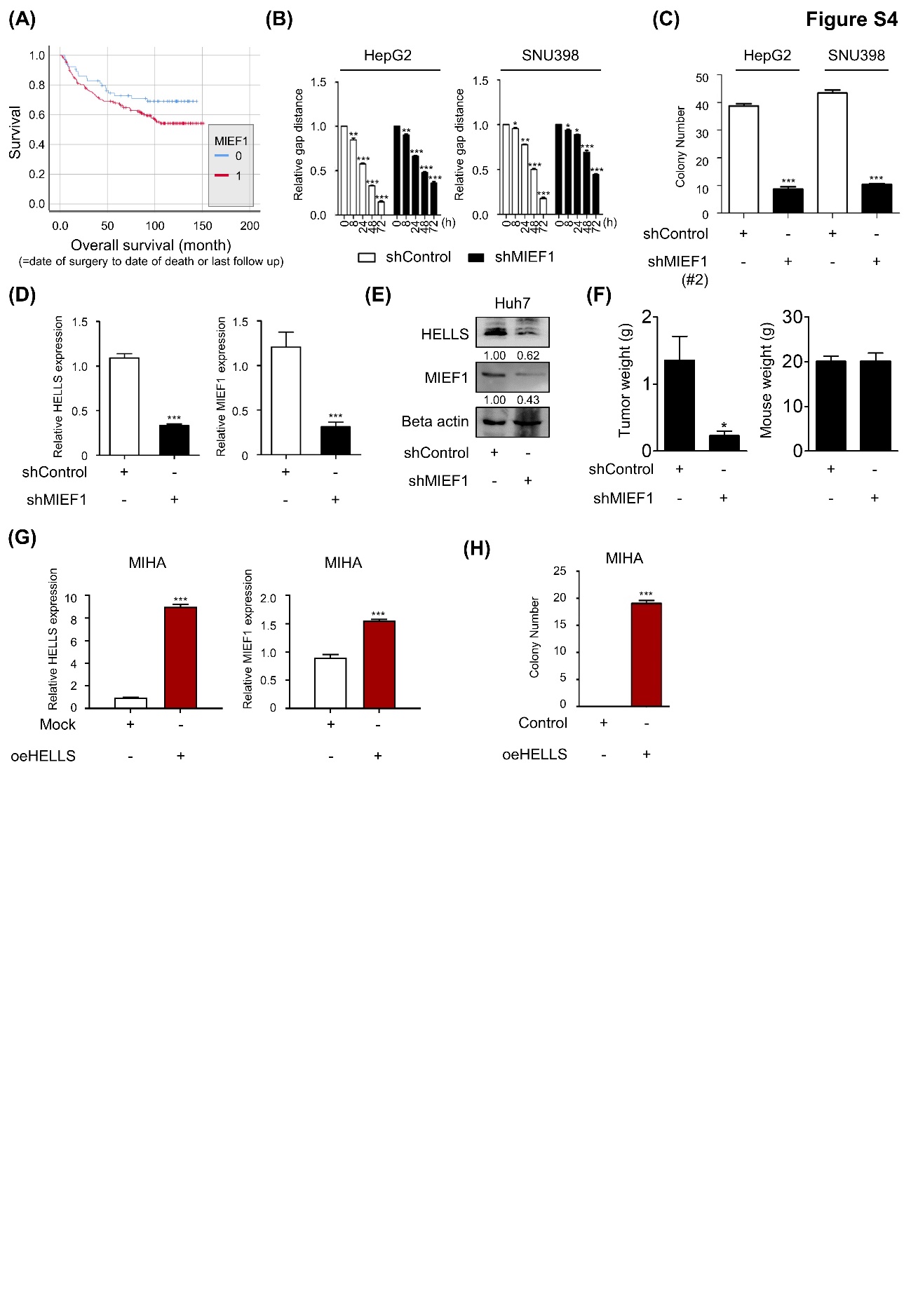
**

**Figure S4. MIEF1 plays an oncogenic role in liver cancer.**

1. Recurrence-free survival time dependent on MIEF1 expression (0: MIEF1 negative, 1: MIEF1 positive).
2. Cell migration rates measured by wound healing assay in HepG2 and SNU398 cells upon MIEF1 loss (mean ± S.E.M., *p < 0.05, **p < 0.01, ***p < 0.001, n=3).
3. Colony formation measured by soft agar assay in HepG2 and SNU398 cells upon HELLS loss (mean ± S.E.M., ***p < 0.001, n=3).
4. Expression levels of HELLS and MIEF1 in Huh7 cells following MIEF1 knockdown, as determined by qRT-PCR (mean ± S.E.M., ***p < 0.001, n=3).
5. Protein levels of HELLS, MIEF1 and Beta actin upon MIEF1 depletion in Huh7 cells were measured using Western blots.
6. Tumor weight and mouse weight in xenograft model. Tumor weight and mouse weight were measured using nude mice injected with Huh7 shControl and shMIEF1 cells (n = 4 per group) and presented as mean ± S.E.M. (*p < 0.05).
7. Expression levels of HELLS and MIEF1 in MIHA cells following MIEF1 overexpression, as determined by qRT-PCR (mean ± S.E.M., ***p < 0.001, n=3).
8. Colony formation measured by soft agar assay in MIHA cells upon MIEF1 overexpression (mean ± S.E.M., ***p < 0.001, n=3).

**
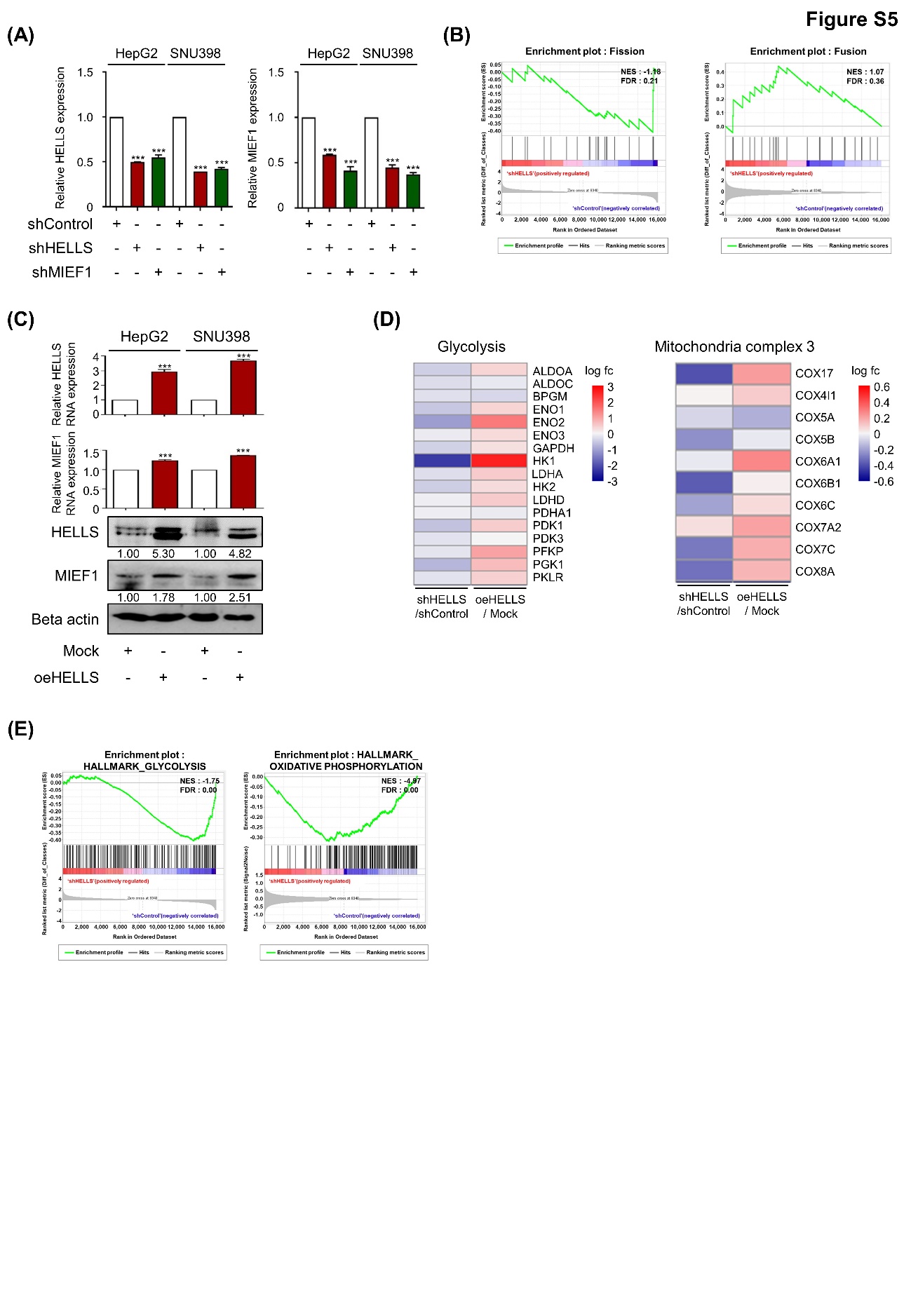
**

**Figure S5. The HELLS-MIEF1 axis regulates mitochondria dynamics and metabolism.**

1. Expression levels of HELLS and MIEF1 in HepG2 and SNU398 cells following HELLS or MIEF1 knockdown, as determined by qRT-PCR (mean ± S.E.M., ***p < 0.001, n=3).
2. Gene set enrichment analysis (GSEA) of HELLS depletion. NES (Normalized Enrichment Score), FDR (FDR q-value).
3. Expression levels of HELLS and MIEF1 in HepG2 and SNU398 cells following HELLS overexpression, as determined by qRT-PCR (mean ± S.E.M., ***p < 0.001, n=3).
4. Relative expression levels of glycolysis and mitochondrial complex 3 gene set in HepG2 cells upon HELLS depletion or overexpression.
5. Gene set enrichment analysis (GSEA) of HELLS depletion. NES (Normalized Enrichment Score), FDR (FDR q-value).

**
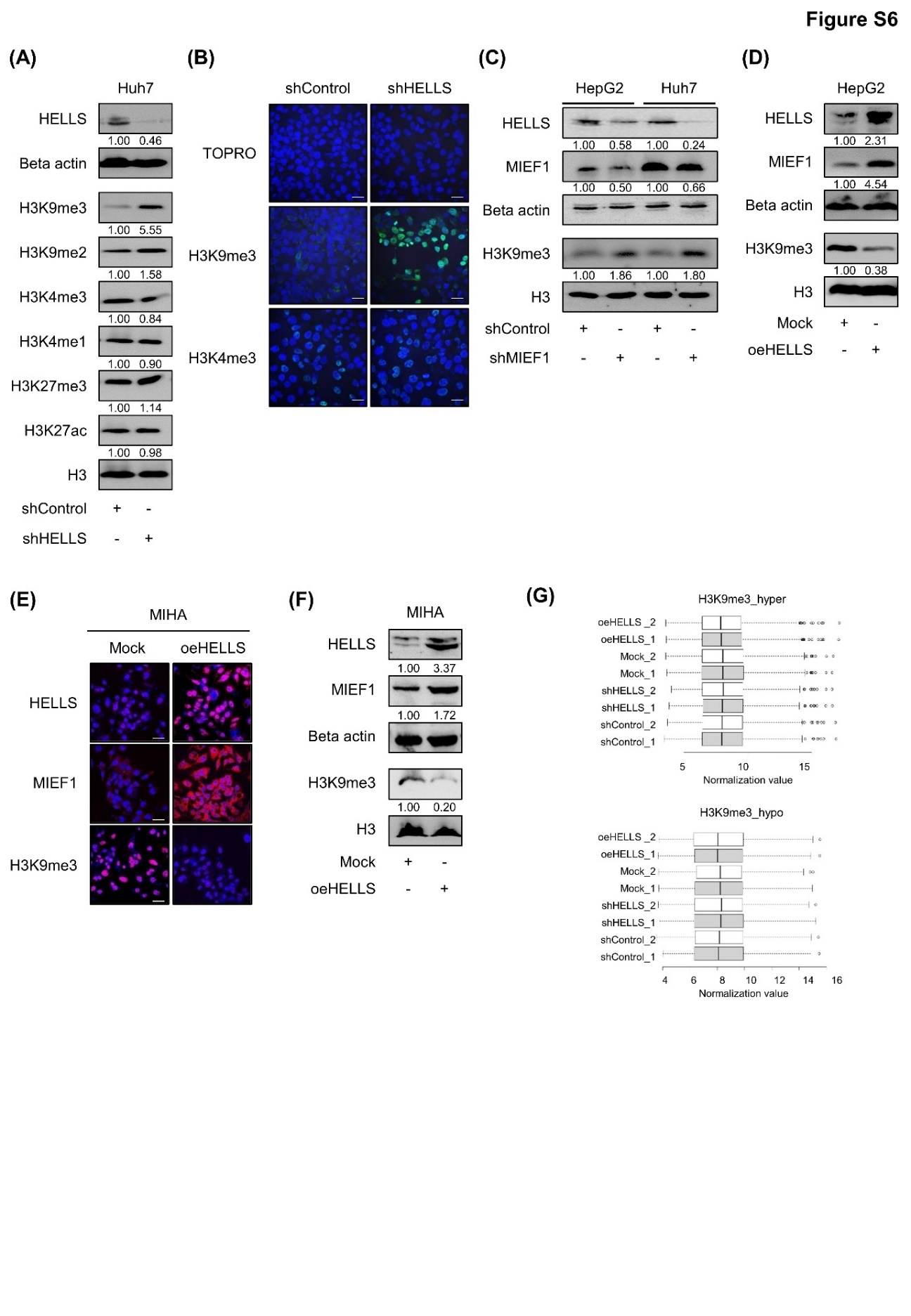
**

**Figure S6.** **Loss of HELLS increases H3K9me3.**

1. Protein levels of HELLS, various histone modifications, and H3 upon HELLS depletion in Huh7 were measured using Western blots.
2. Representative confocal images of H3K9me3, H3K4me3, and TORP upon HELLS depletion. The scale bar represents 100 μm.
3. Protein levels of HELLS, MIEF1, Beta actin, H3K9me3, and H3 upon MIEF1 depletion in HepG2 and Huh7 were measured using Western blots.
4. Protein levels of HELLS, MIEF1, Beta actin, H3K9me3, and H3 upon HELLS overexpression in HepG2 were measured using Western blots.
5. Representative confocal images of HELLS, MIEF1, and H3K9me3 upon HELLS overexpression in MIHA. The scale bar represents 100 μm.
6. Protein levels of HELLS, MIEF1, Beta actin, H3K9me3, and H3 upon HELLS overexpression in MIHA were measured using Western blots.
7. Gene expression levels from microarray analysis of genes associated with hypermethylated and hypomethylated H3K9me3 peaks in HepG2 cells.

**
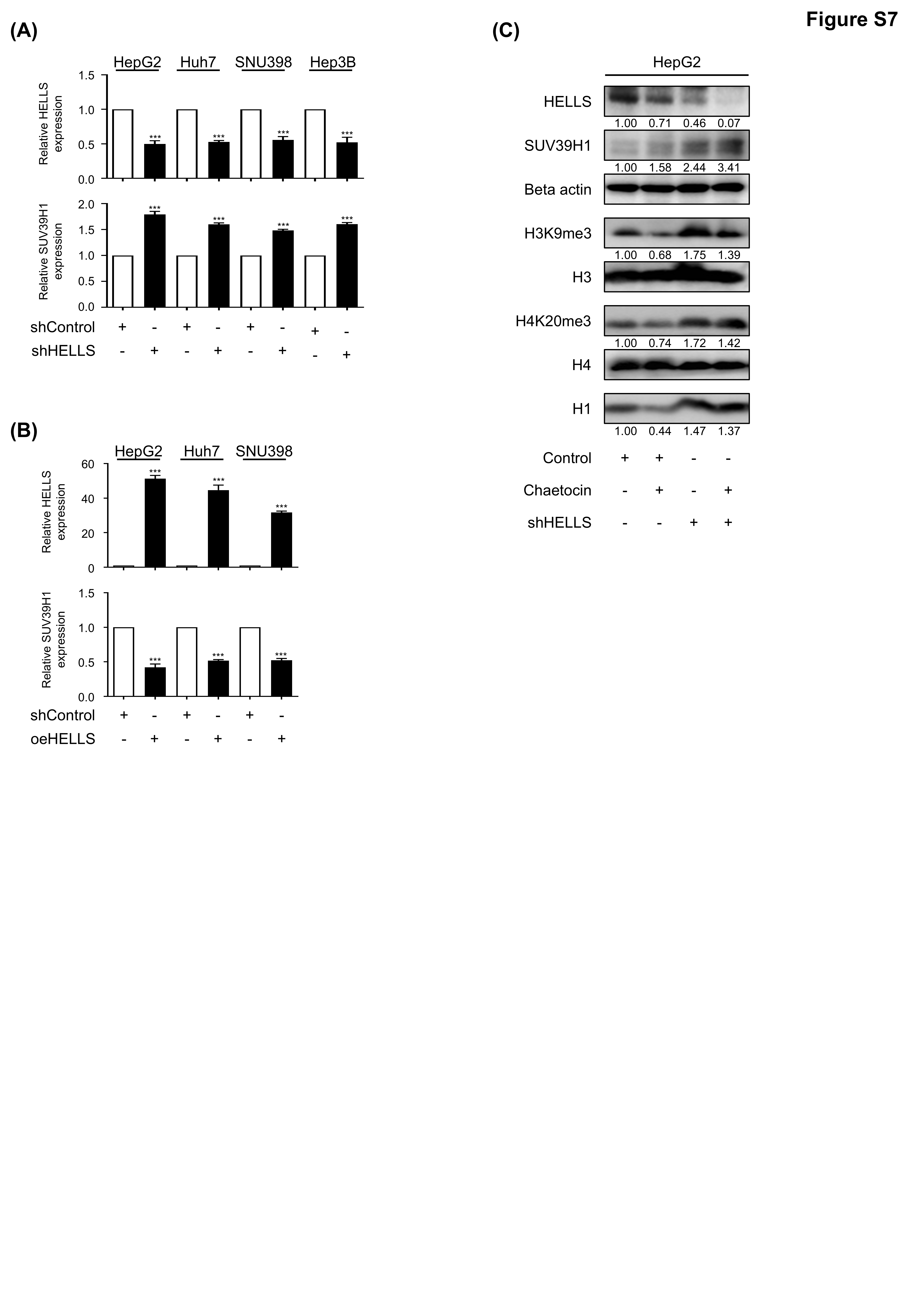
**

**Figure S7. Loss of HELLS induces overall H3K9 hypermethylation.**

1. Expression levels of HELLS and SUV39H1 in HepG2, Huh7, SNU398, and Hep3B cells following HELLS knockdown, as determined by qRT-PCR (mean ± S.E.M., ***p < 0.001, n=3).
2. Expression levels of HELLS and SUV39H1 in HepG2, Huh7, and SNU398 cells following HELLS overexpression, as determined by qRT-PCR (mean ± S.E.M., ***p < 0.001, n=3).
3. Protein levels of HELLS, SUV39H1, Beta actin, H3K9me3, H3, H4K20me3, H4, and H1 were measured by Western blot analysis in Control, chaetocin-treated, shHELLS, and shHELLS + chaetocin-treated HepG2 cells.

**
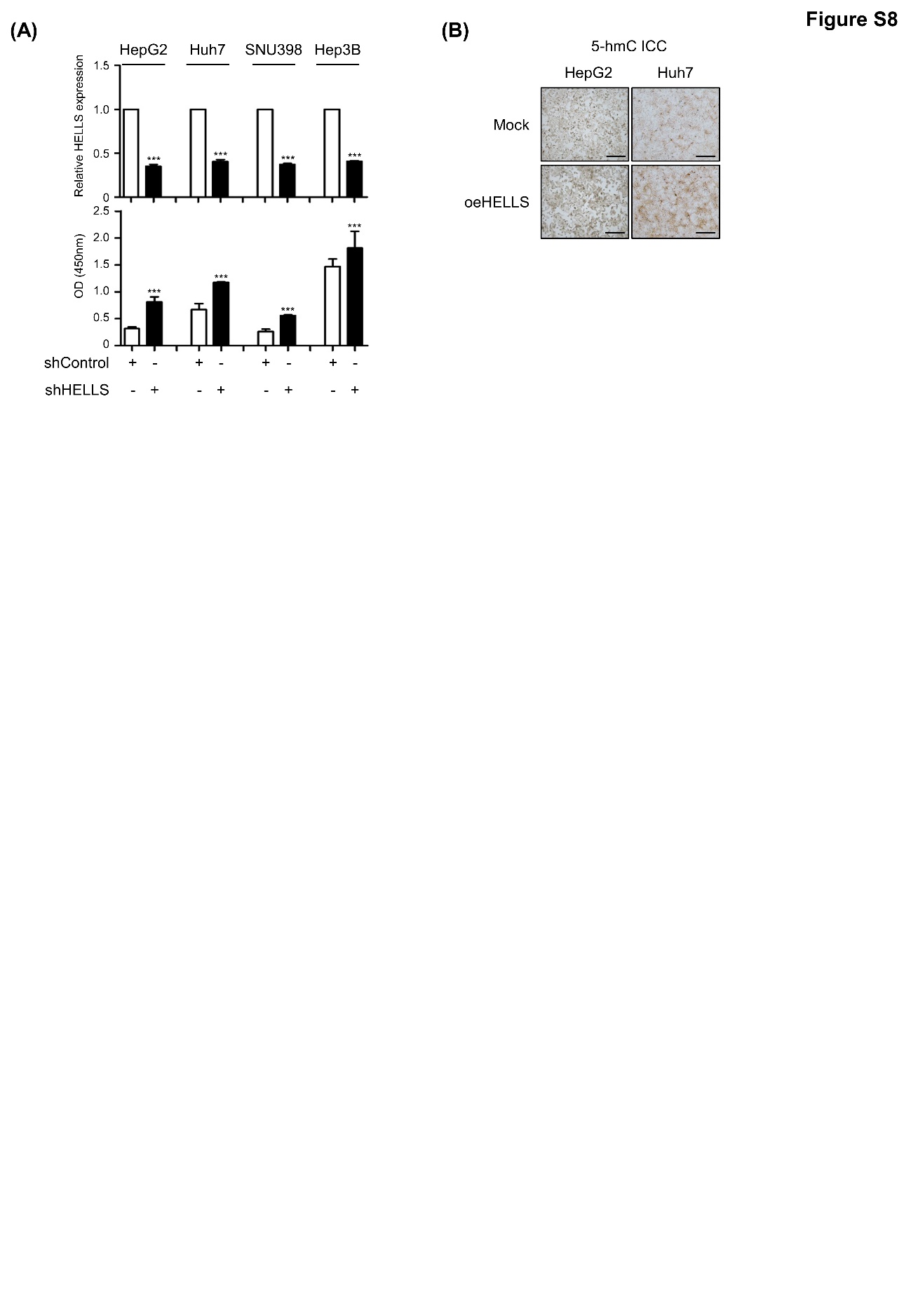
**

**Figure S8. Loss of HELLS induces global DNA hypermethylation.**

1. Expression levels of HELLS and global DNA methylation levels in HepG2, Huh7, SNU398, and Hep3B cells following HELLS knockdown, as determined by qRT-PCR (mean ± S.E.M., ***p < 0.001, n=3).
2. Representative ICC staining for 5hmC upon HELLS overexpression in HepG2 and Huh7 (Scale bars = 100 μm).

**
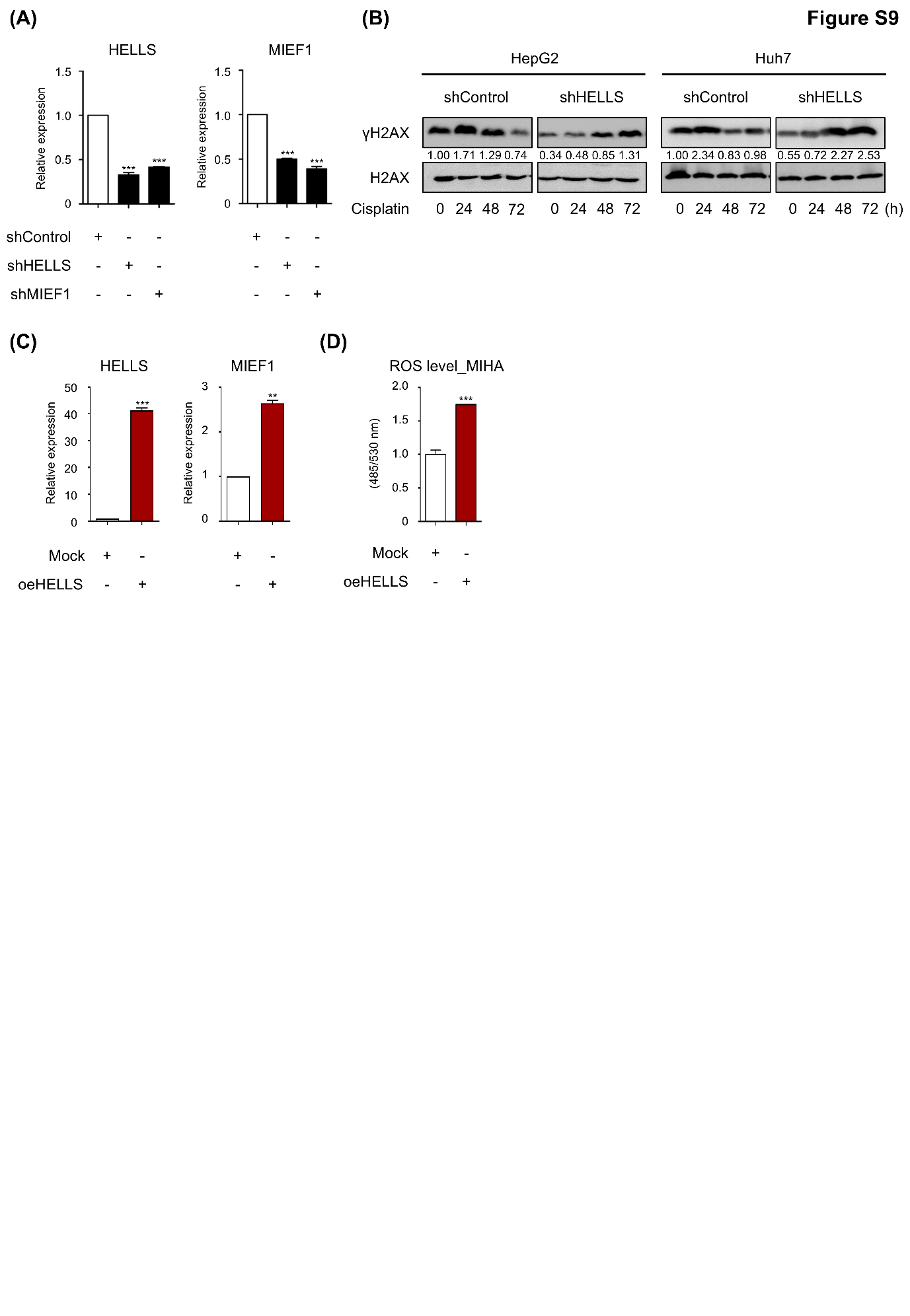
**

**Figure S9. Suppression of the HELLS-MIEF1 axis reduces DNA damage.**

1. Expression levels of HELLS and MIEF1 in HepG2 following HELLS or MIEF1 knockdown, as determined by qRT-PCR (mean ± S.E.M., ***p < 0.001, n=3).
2. Protein levels of γH2AX and H2AX following HELLS knockdown and cisplatin treatment in HepG2 and Huh7 cells were measured using Western blots.
3. Expression levels of HELLS and MIEF1 in HepG2 following HELLS overexpression, as determined by qRT-PCR (mean ± S.E.M., **p < 0.01, ***p < 0.001, n=3).
4. ROS levels upon HELLS overexpression in MIHA (mean ± S.E.M., ***p < 0.001, n=3).
5. Scheme of the HELLS-MIEF1 axis.


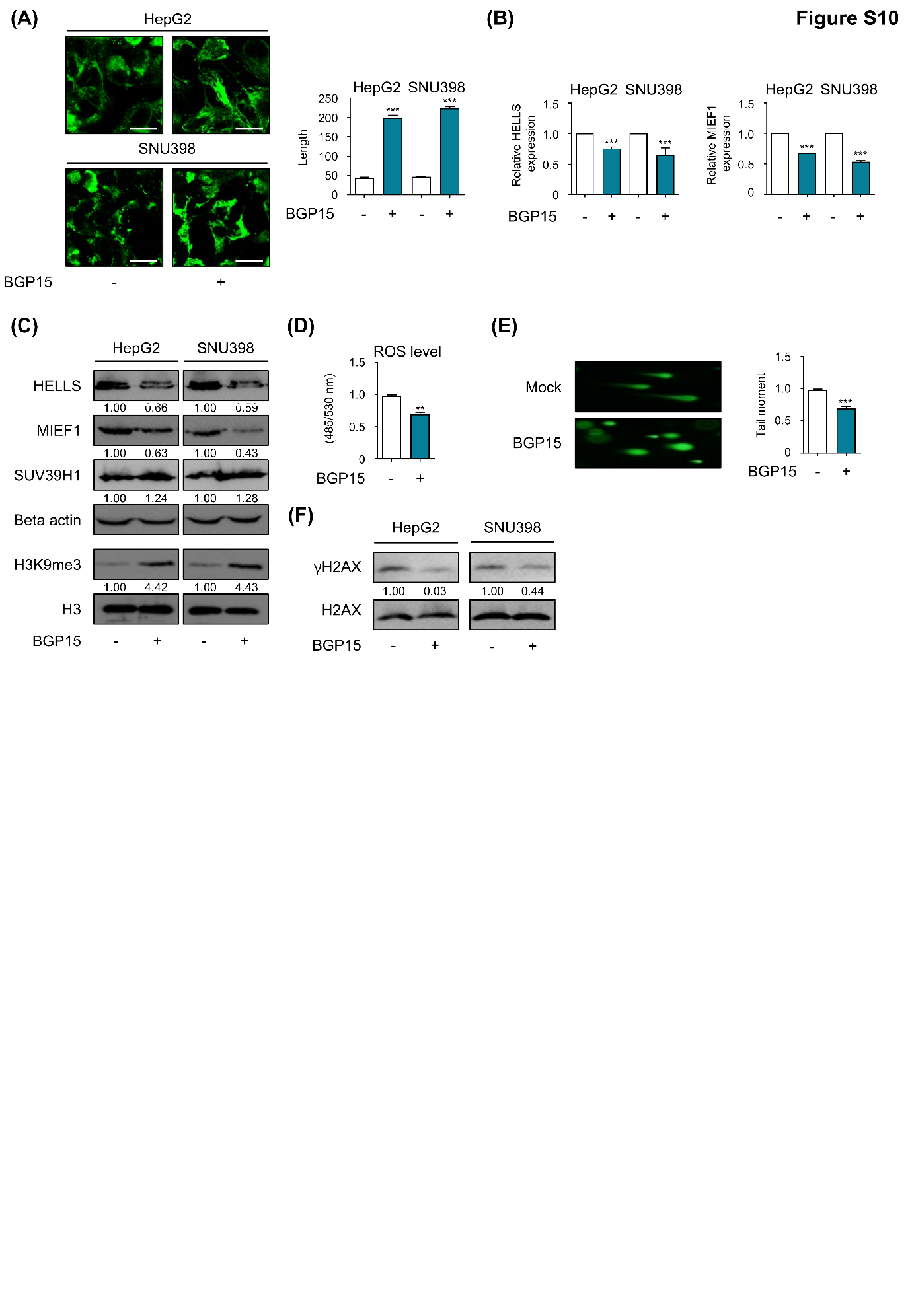


**Figure S10. BGP15-induced mitochondrial fusion enhances genomic stability in HCC.**

1. Representative confocal images of mitochondria in HepG2 and SNU398 cells following BGP15 treatment. Cells were transfected with mt-ro2GFP. The scale bar represents 20 μm. Quantification of mitochondrial length was measured (mean ± S.E.M., ns: non-significant, ***P < 0.001, n=3).
2. The relative mRNA levels of HELLS and MIEF1 in HepG2 and SNU398 cells following BGP15 treatment were presented as mean ± S.E.M. ( ***p < 0.001, n=3).
3. Protein levels of HELLS, MIEF1, SUV39H1, Beta actin, H3K9me3, and H3 in HepG2 and SNU398 cells following BGP15 treatment were measured using western blots.
4. ROS levels in HepG2 following BGP15 treatment (mean ± S.E.M., **p < 0.01, n=3).
5. Representative comet images stained with a green DNA staining solution and bar graph showing the quantification of tail moment in HepG2 cells following BGP15 treatment (mean ± S.E.M., ***p < 0.001, n=3).
6. Protein levels of γH2AX and H2AX in HepG2 and SNU398 cells following BGP15 treatment were measured using western blots.

**Supplementary Tables**

**Table S1. Primer sequence for RT-PCR. F: Forward primer. R: reverse primer**

| **Gene** | **Primer sequence** |
| --- | --- |
| **Beta actin** | F: 5’-GAGTCAACGGATTTGGTCGT-3 |
|  | R: 5’-TGGAAGATGGTGATGGGATT-3 |
| **HELLS** | F: 5’-GTGGCCCTTTGTCTACACTTCC-3 |
|  | R: 5’-TCGGTCTCTCATGGCTATTTCA-3 |
| **MIEF1** | F: 5’-CAGAAGCCCTCACACTGGAG-3 |
|  | R: 5’-CCGCCACAGGTTGTCATACT-3 |
| **SUV39H1** | F: 5’-TCGCAAGAACAGCTTCGTCA-3 |
|  | R: 5’-CACGGTGTACACGTCCTCCA-3 |

**Table S2. Primer sequence for ChIP-PCR. F: Forward primer. R: reverse primer**

| **Gene** | **Primer sequence** |
| --- | --- |
| **MIEF1 proximal promoter** | F: 5’-CCTCTGAACTTCTGCCAGCA -3 |
|  | R: 5’-CCGGTCTTCTCCAAGCTCTC-3 |
| **SUV39H1 proximal promoter** | F: 5’-TACGGCACTAGTCCACCTGG -3 |
|  | R: 5’-CGCTGGTCTGTCTGTCACAC-3 |

**Table S3. Antibody list for western blots, ChIP, and ICC**

|  | **Antibody name** | **Company** | **Catalog #** |
| --- | --- | --- | --- |
| **Western blots** | **HELLS** | Bethyl Laboratories | A300-226A |
|  | **MIEF1** | proteintech | 20164-1-AP |
|  | **SUV39H1** | millipore | 05-615 |
|  | **Beta actin** | Cell Signaling Technology | 4967s |
|  | **H3K9me3** | active motif | 39161 |
|  | **H3K4me3** | active motif | 39159 |
|  | **H4K20me3** | Abcam | ab195479 |
|  | **H3K9me2** | millipore | 17-681 |
|  | **H3K27me3** | merk | 07-449 |
|  | **H3K27ac** | Abcam | ab177178 |
|  | **H3K4me1** | Abcam | ab8895 |
|  | **H1** | Abcam | ab125027 |
|  | **H3** | Abcam | ab1791 |
|  | **H4** | Abcam | ab7311 |
|  | **pho-AMPK** | Cell Signaling Technology | 2535 |
|  | **AMPK** | Cell Signaling Technology | 2532 |
|  | **γH2AX** | Abcam | ab11174 |
|  | **H2AX** | Cell Signaling Technology | 2595 |
| **ChIP** | **HELLS** | Bethyl Laboratories | A300-226A |
|  | **H3K4me3** | active motif | 39159 |
| **ICC** | **HELLS** | Bethyl Laboratories | A300-226A |
|  | **MIEF1** | proteintech | 20164-1-AP |
|  | **H3K9me3** | active motif | 39161 |
|  | **H3K4me3** | active motif | 39159 |
|  | **5hmc** | active motif | 39791 |
|  | **Rhodamine Red-X-AffiniPure Goat Anti-Rabbit IgG (H+L)** | Jackson Immuno Research | 111-295-144 |
