# Supplementary material for: HELLS controls mitochondrial dynamics and genome stability in liver cancer by collusion with MIEF1
Source: Cell Death Dis. 2025 Apr 2;16(1):239. doi: 10.1038/s41419-025-07589-x (PMC11965466; doi:10.1038/s41419-025-07589-x)

Original western blots

Figure S11

Figure1. D

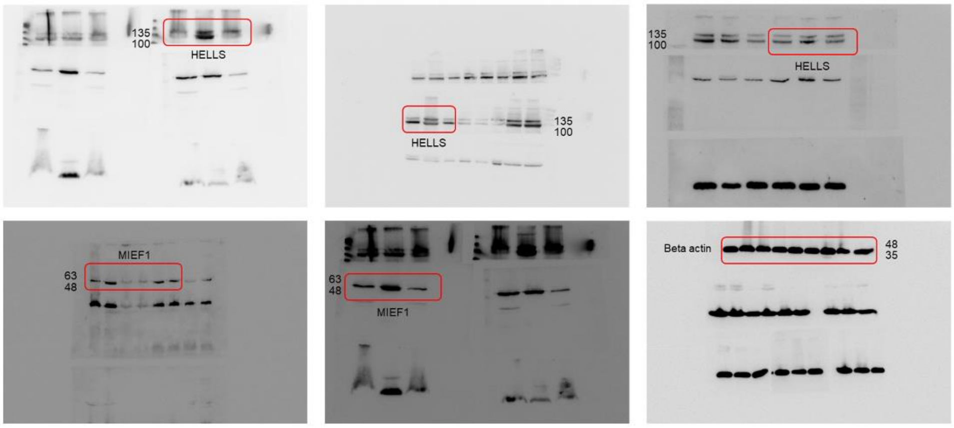

Figure1. E

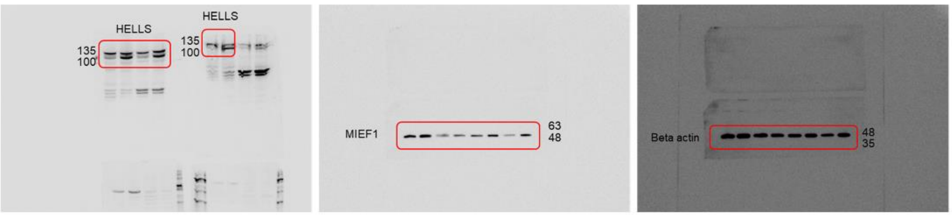

Figure2. D

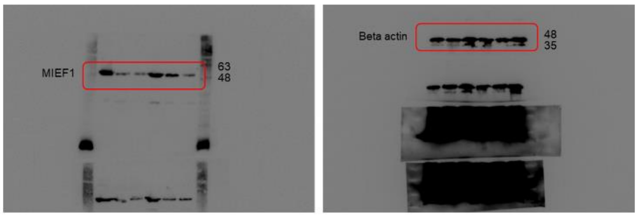

Figure3. H

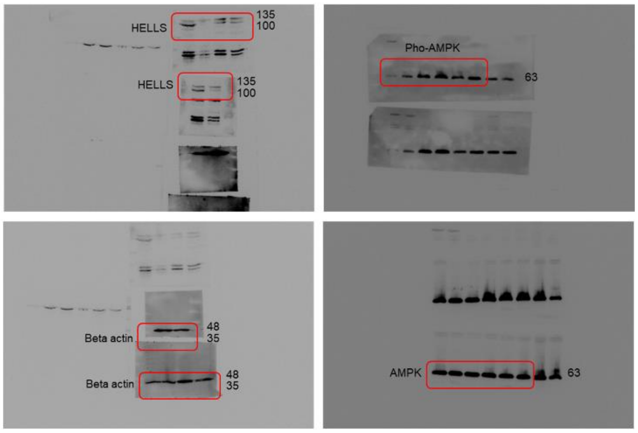

Figure4. A

Figure S12

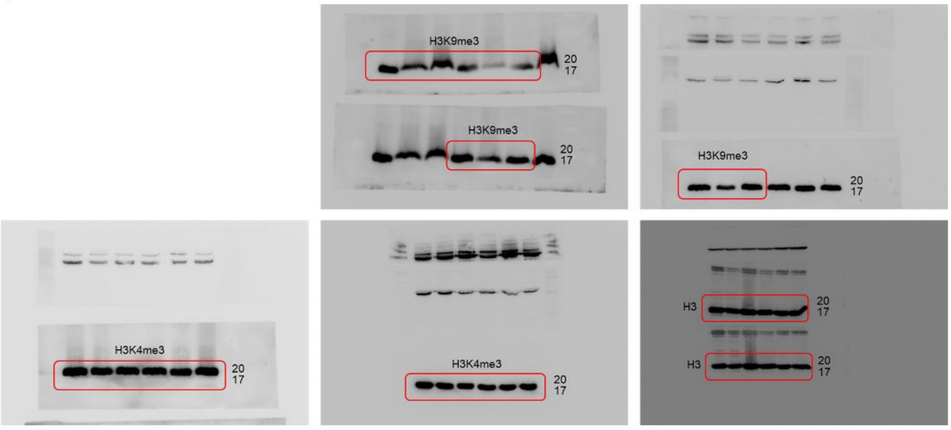

Figure4. G

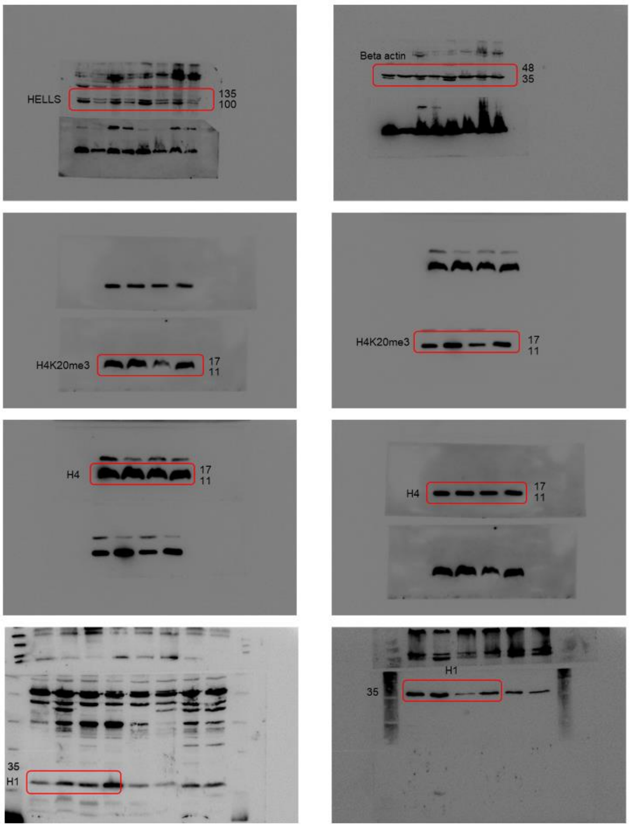

Figure5. B

Figure S13

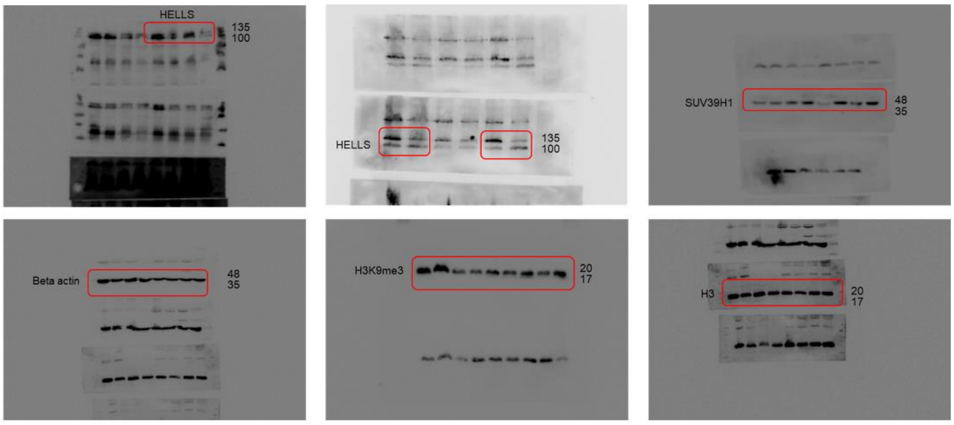

Figure5. C

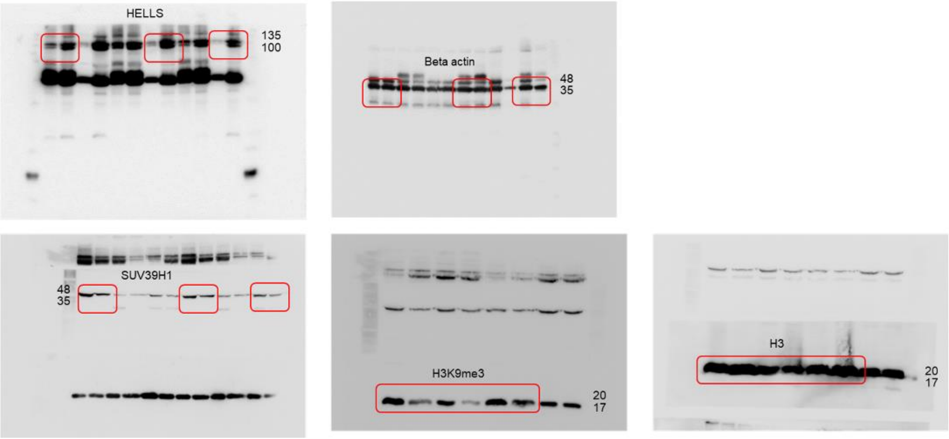

Figure6. B

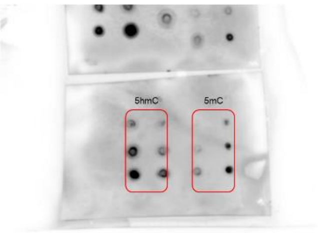

Figure7. C

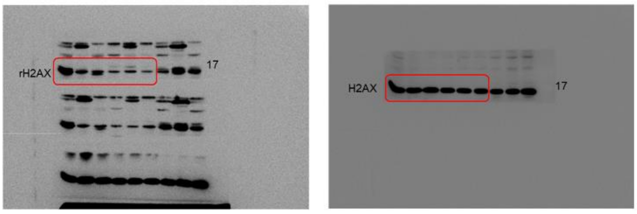

Figure S14

Figure8. C

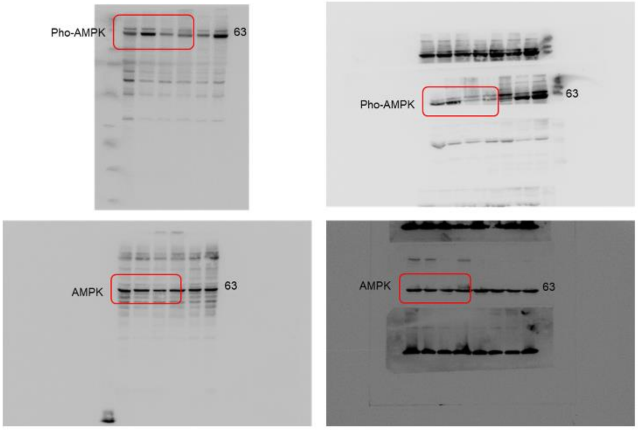

Figure8. D

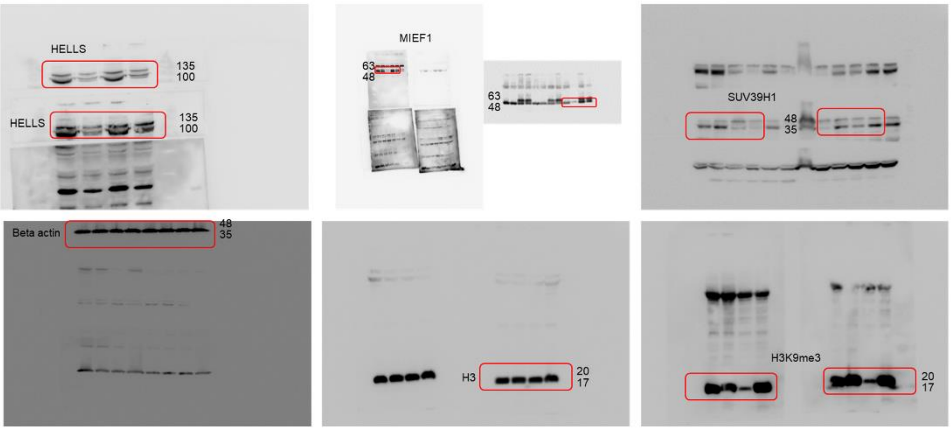

Figure8. G

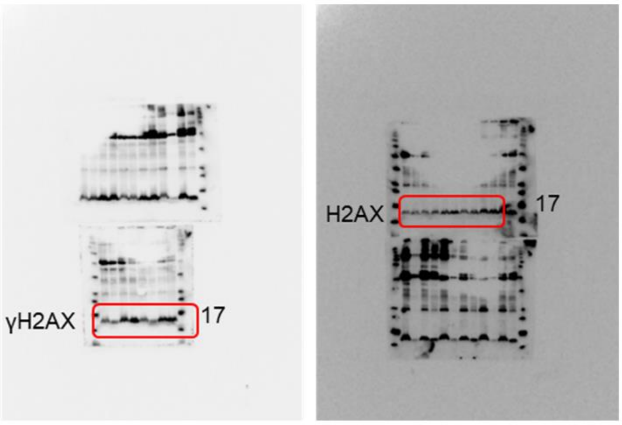

Supple1. D

Figure S15

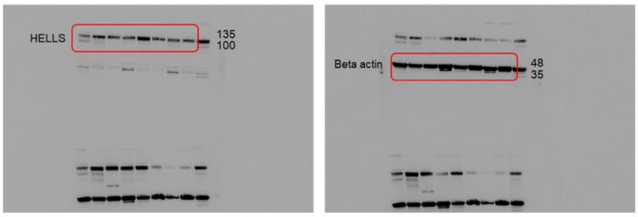

Supple1. E

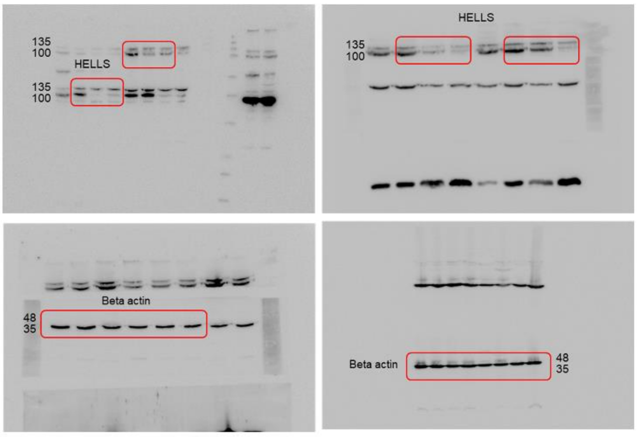

Supple4. E

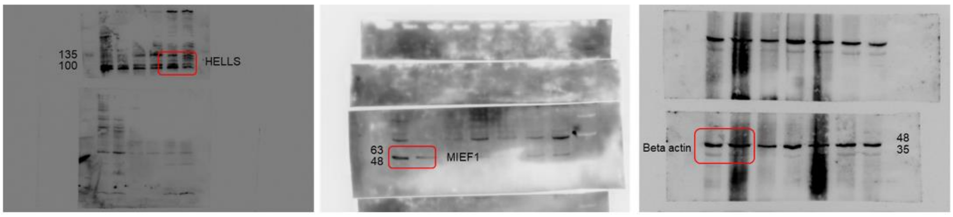

Supple5. C

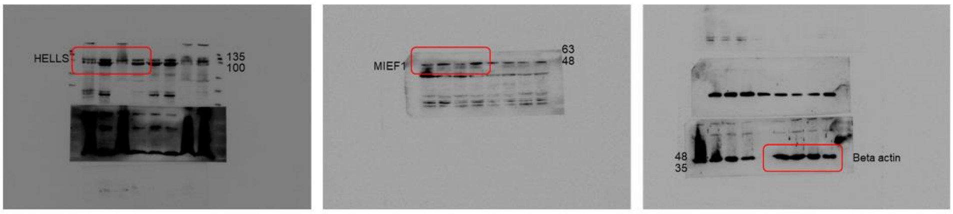

Figure S16

Supple6. A

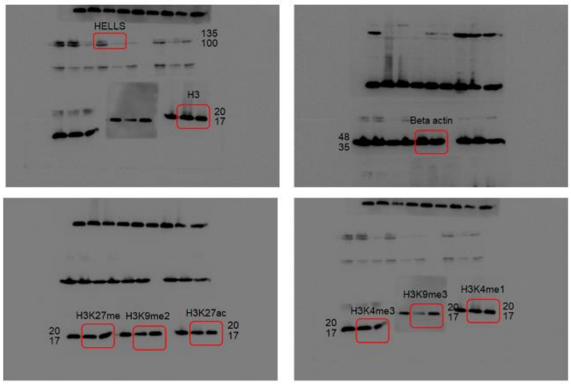

Supple6. C

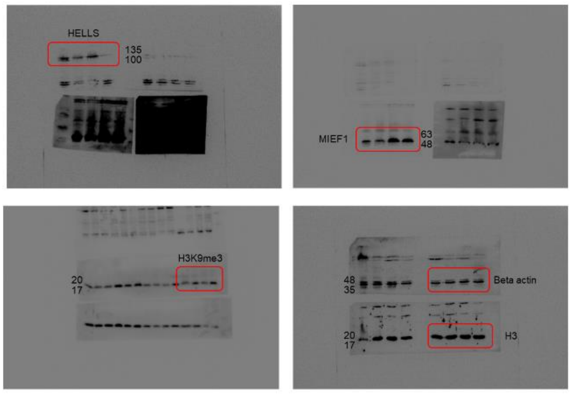

Supple6. D

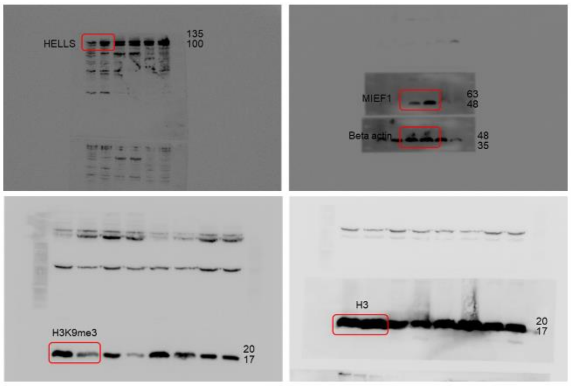

Supple6. F

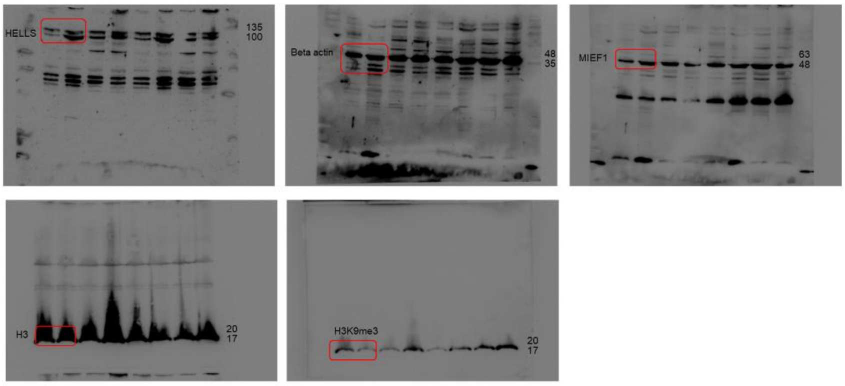

Figure S17

Supple7. C

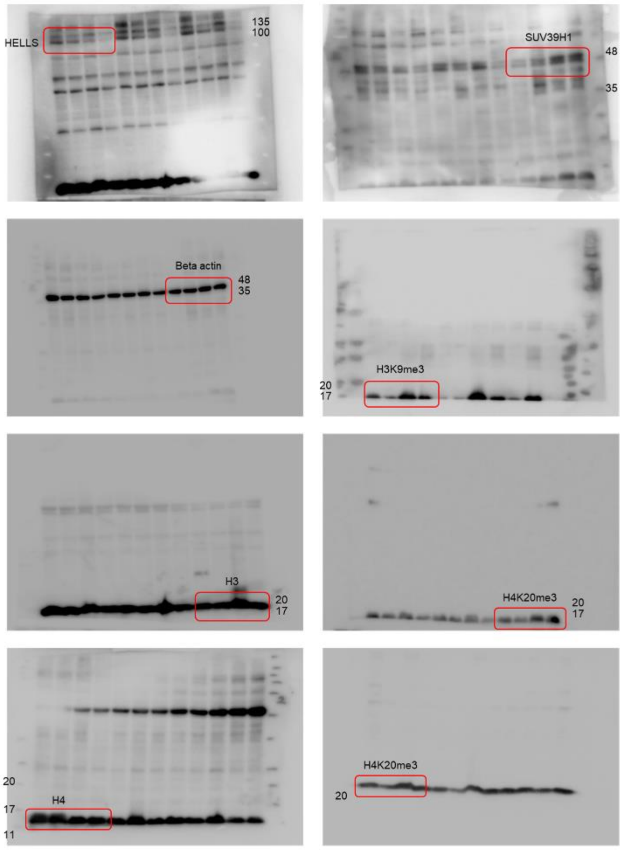

Supple9. B

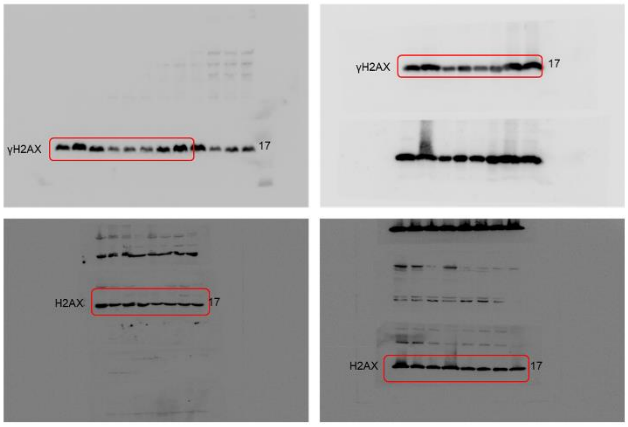

Figure S18

Supple10. C

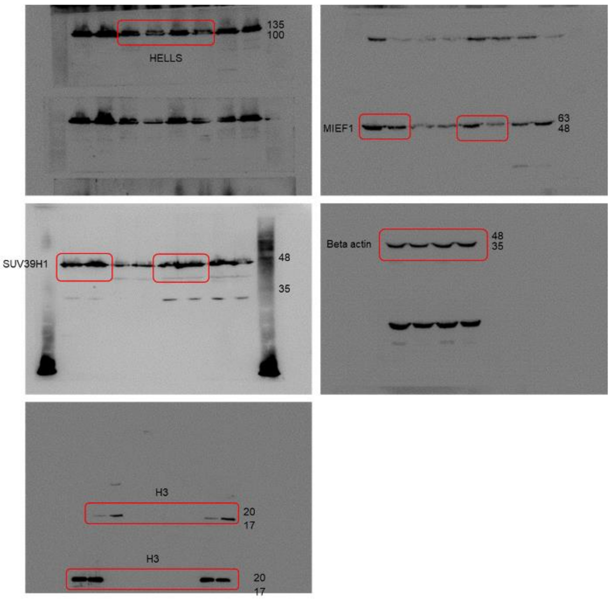

Supple10. D

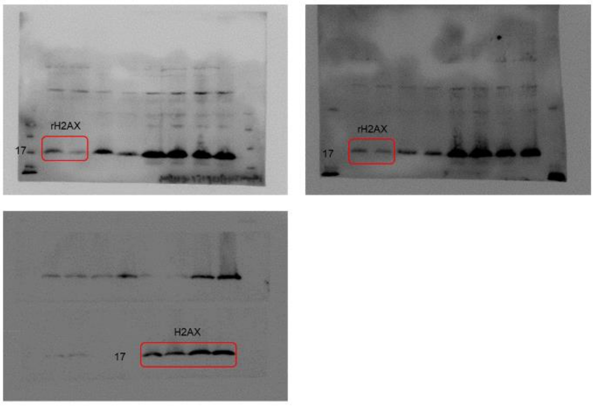

Supplement: Supplementary file 2 — supplementary file_Original western blots [file 41419_2025_7589_MOESM2_ESM.pdf]
